# High‐Efficiency, Long‐Lifetime and Color‐Tunable Hybrid WOLEDs Using a Platinum Complex with Voltage‐Dependent Monomer and Aggregate Emission

**DOI:** 10.1002/advs.202411364

**Published:** 2025-01-21

**Authors:** Yangyang Xin, Mao Mao, Shuo Xu, Kaixin Tan, Gang Cheng, Hai Zhang, Hengyi Dai, Tianyu Huang, Dongdong Zhang, Lian Duan, Chi‐Ming Che

**Affiliations:** ^1^ Key Laboratory of Organic Optoelectronics and Molecular Engineering of Ministry of Education Department of Chemistry Tsinghua University Beijing 100084 P. R. China; ^2^ State Key Laboratory of Synthetic Chemistry Department of Chemistry HKU‐CAS Joint Laboratory on New Materials The University of Hong Kong Pokfulam Road Hong Kong SAR 999077 P. R. China; ^3^ HKU Shenzhen Institute of Research and Innovation Shenzhen Guangdong 518053 P. R. China; ^4^ Hong Kong Quantum AI Lab Limited Unit 909‐915 of 17W Building, Science Park Hong Kong SAR P. R. China

**Keywords:** aggregate emission, color‐tunable, dual emissive layer, platinum complex, WOLED

## Abstract

Color‐tunable white organic light‐emitting diodes (CT‐WOLEDs) have attracted widespread attention given their large color variation to meet the different daily scenarios from the perspective of circadian rhythm. However, most reported CT‐WOLEDs, especially the tri‐color devices, exhibit poor performances and sophisticated structures. Here, a simple structure tri‐color CT‐WOLED is demonstrated that simultaneously exhibits high efficiency, ultralong operation lifetime, and wide color‐tunable range for dynamic sunlight emulation. The design is based on a newly developed platinum complex that can emit light efficiently in both monomer and aggregation states, providing voltage‐dependent green‐to‐red phosphorescence emission, not only ensuring tunable colors in WOLEDs but also simplifying the device structure. Combining with a blue delayed fluorescence material, this hybrid device exhibits a wide range of tunable colors with Commission Internationale de l’Eclairage 1931 (CIE) coordinates and correlated color emperature (CCT)  shifts from (0.502, 0.474) and 2628 K at 2.6 V to (0.211, 0.334) and 14860 K at 8 V, achieving good visual alignment with sunlight color throughout the day. This same device also shows high external quantum efficiencies from 28.8% at maximum to 26.2% at 5000 cd m^−2^. Furthermore, an impressively long time of 21,144 h is achieved to decay to 90% of the initial luminance at 100 cd m^−2^, being the longest among recorded CT‐WOLEDs.

## Graphical abstract

The implementation of a tri‐color hybrid color‐tunable white OLED (CT‐WOLEDs) is demonstrated with blue delayed fluorescence and voltage‐dependent green‐to‐red phosphorescence, showing high efficiency, ultralong operation lifetime, and wide color‐tunable range. The CT‐WOLEDs exhibit good visual alignment with full‐day sunshine. This work provides a feasible strategy for CT‐WOLEDs and paves the way for commercialization.

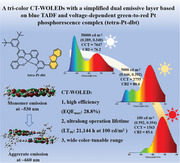

## 1 Introduction

White organic light‐emitting diodes (WOLEDs) can be made into highly efficient, flexible, large‐area light sources, and their ultrathin thickness also provides applications in various environments.^[^ ^1^ ^]^ So WOLED is considered to have broad application prospects in the future solid‐state lighting market. In addition to the above advantages, in order to meet different daily scenarios from the perspective of circadian rhythm, a qualified WOLED should also emit bright cool‐light with a high correlated color temperature (CCT) to improve people's activity and alertness, while dim warm‐light with low CCT is thought to keep humans healthy at night.^[^ ^2^ ^]^ Under this circumstance, color‐tunable WOLEDs (CT‐WOLEDs) therefore emerged and attracted widespread attention.^[^ ^3^ ^]^ Given the advantages of organic emitters with easily tunable colors covering the entire visible region, CT‐WOLEDs have proven to be promising candidates for physiologically friendly, voltage‐dependent color‐tunable lighting devices. Several conceptually diverse device structures have been constructed and evaluated to achieve the desired electroluminescence (EL) properties.

An ideal CT‐WOLED should satisfy both a wide range of color changes from warm to cool‐light and a high efficiency in a large luminance region concurrently. The most straightforward strategy to construct such a device should be a parallel^[^ ^4^ ^]^ or series structure of independently controlled sub‐OLED arrays^[^ ^5^ ^]^ with different voltage operation requirements. However, despite the broad color‐tuning region, this strategy is limited by sophisticated device structures. Alternatively, voltage‐dependent activation of multiple emitters in a single‐cell device can be considered a simpler strategy^[^ ^6^ ^]^ but faces challenges in terms of color adjustability. By exploring the voltage‐dependent energy transfer process from a blue emitter to a yellow emitter (fBu‐Au‐CN) with a long‐lived emissive excited state, which can be saturated at high voltages to prevent energy transfer, CT‐WOLEDs with Commission Internationale de l’Eclairage 1931 (CIE) coordinates from (0.40,0.50) at 5 V to (0.20,0.35) at 10 V can be obtained, but their maximum external quantum efficiency (EQE_max_) is low (13.16%) and shows severe efficiency roll‐off.^[^ ^7^ ^]^ Fluorescent CT‐WOLEDs with thermally activated delayed fluorescent (TADF) sensitized emissions have also been reported, which rely on hole‐trapping induced recombination zone shift to adjust color, with CIE coordinates ranging from (0.40, 0.47) at 100 cd m^−2^ to (0.27, 0.33) at 5000 cd m^−2^. At an initial luminance (*L_0_*) of 100 cd m^−2^, the EQE_max_ is 30.7% and the LT_80_ exceeds 20 000 h.^[^ ^8^ ^]^ Despite significant progress, CT‐WOLEDs still face challenges. First, the reported device still relies on dual‐color emission, sacrificing color index. Although the tri‐color device has better performance, its application is still affected by the sophisticated device structure. Second, device performance still needs to be improved, especially operational stability. In addition, other more feasible color adjustment strategies need to be developed.

Noteworthy, color‐tunable devices based on single‐molecule platinum complexes have also emerged in recent years.^[^ ^9^ ^]^ Due to the planar molecular structure, Pt(II) complexes tend to form aggregates, resulting in dual emissions, including monomer emission at high energy wavelengths and aggregation emission at lower energy wavelengths.^[^ ^10^ ^]^ A tetradentate Pt(II) complex named **tetra‐Pt‐dbf** can effectively emit light in the monomer and aggregation states, achieving a large color‐tuning span (red to yellowish green) with high efficiency and long‐term lifetime (>520 000 h at 100 cd m^−2^).^[^ ^11^ ^]^ However, efforts to fabricate single‐molecular CT‐WOLEDs based on Pt complexes face considerable challenges. Until now, the CIE coordinates for such devices can only be adjusted from (0.47, 0.44) at 3 V to (0.36, 0.48) at 11 V, with a color rendering index of 82 and an EQE of up to 20.75%.^[^ ^12^ ^]^ However, the emission color range is quite small, and the device's operational stability is severely poor. Interestingly, green‐to‐red light‐emitting Pt complexes may provide the opportunity to fabricate tri‐color CT‐WOLEDs with simplified device structures by introducing blue emission components.

Here, we demonstrate the implementation of tri‐color hybrid CT‐WOLEDs based on blue delayed fluorescence and voltage‐dependent green‐to‐red phosphorescence from a single Pt complex, simultaneously achieving high efficiency, ultralong operational lifetime, and wide color tuning range. Following our previous color‐tunable Pt complex (**tetra‐Pt‐dbf**), we introduced a sulfur atom to replace oxygen and prepare **tetra‐Pt‐dbt**, which has a larger spin‐orbital coupling (SOC) for a faster radiative decay and higher photo‐luminance quantum yield (PLQY). Furthermore, **tetra‐Pt‐dbt** can emit light efficiently in both monomer and aggregation states to provide voltage‐dependent green‐to‐red phosphorescence emission in devices. A simplified dual‐emissive‐layer WOLED with a blue TADF layer and a Pt layer inherits the compound's color‐tunable capabilities, showing a broad color‐tuning range with CIE coordinates and CCT from (0.502, 0.474) and 2628 K at 2.6 V to (0.211, 0.334) and 14 860 K at 8V. Furthermore, the maximum external quantum efficiency is high, ranging from 28.8% at 378 cd m^−2^ to 26.2% at 5000 cd m^−2^. An impressively long LT_90_ (time to decay to 90% of the initial luminance) of over 21144 h was obtained at 100 cd m^−2^ luminance, the longest reported among CT‐WOLEDs. We further demonstrate the good visual alignment of such device's emitting light in sunlight throughout the day. Our work provides a feasible strategy for CT‐WOLED and paves the way for commercialization, such as the regulation of circadian rhythms in healthcare and dynamic visual sunlight emulation in the field of virtual reality (VR).

## 2 Results and Discussion

### 2.1 Synthesis and Characterization

An ideal Pt‐color‐tunable phosphorescent emitter should simultaneously satisfy high PLQY in both monomer and aggregate states, fast radiative decay rate with short‐excited state lifetime, high operational stability and wide color‐range, which all face formidable challenges. Here, molecular design draws on our previous work.^[^ ^13^ ^]^ Starting from Pt‐X‐3, the first compound that showed voltage‐dependent emission color but limited color‐range, we introduced planar rigid dibenzofuran (DBF) units at the terminal pyridine ring (**tetra‐Pt‐dbf**) to enhance molecular aggregation to achieve a wider color range.^[^ ^11^ ^]^ However, **tetra‐Pt‐dbf** only achieved a moderate PLQY of 0.82 for the monomer in toluene, and its lifetime should also be shortened. To address these issues, we optimized this compound by replacing the DBF unit with a dibenzothiophene (DBS) unit group to produce **tetra‐Pt‐dbt**. The sulfur atoms of DBS can enhance SOC,^[^ ^14^ ^]^ thereby facilitating radiative decay and improving luminescence properties while maintaining a rigid planar structure to achieve strong intermolecular aggregation, thus achieving a wide color‐range.

**Figure 1:**
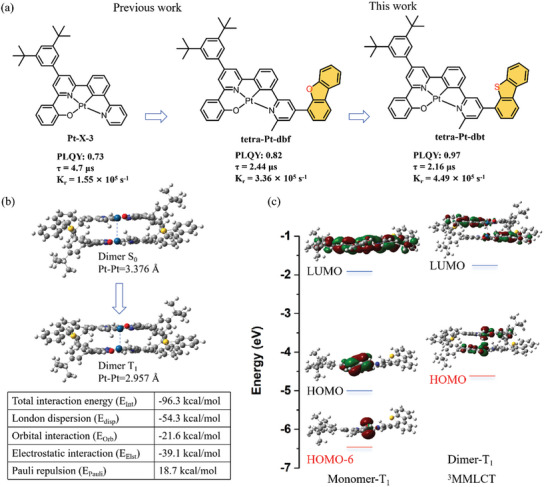
a) Chemical structures and selected performance data of **Pt‐X‐3**, **tetra‐Pt‐dbf** and **tetra‐Pt‐dbt**. b) Calculated Pt‐Pt distance for the [**tetra‐Pt‐dbt**]_2_ in the S_0_ and T_1_ state and the EDA calculation results of the [**tetra‐Pt‐dbt**]_2_ dimer. c) Calculated frontier molecular orbital (MO) diagrams of **tetra‐Pt‐dbt** monomer and dimer using optimized geometries in the T_1_ excited state.

**Figure 2:**
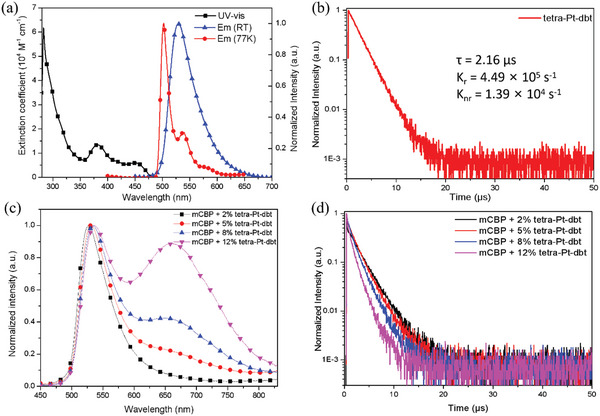
a) UV–vis absorption and emission spectra of **tetra‐Pt‐dbt** in toluene at room temperature and at 77 K. b) Photo‐luminescence decay curves of **tetra‐Pt‐dbt** in toluene after N_2_‐bubbling for 10 min. c) Emission spectra of **tetra‐Pt‐dbt** in mCBP films with different doping concentrations (2 to 12 wt.%). d) Transient PL decay spectra at ≈530 nm of **tetra‐Pt‐dbt** in mCBP films with different doping concentrations (2 to 12 wt.%).

The electronic and photo‐physical properties of **tetra‐Pt‐dbt** were studied through Density Functional Theory (DFT) and Time‐Dependent Density Functional Theory (TD‐DFT) calculations. The absorption spectrum of **tetra‐Pt‐dbt** in toluene solution calculated using the optimized structure is similar to the experimental result (Figure 2; Figure S1, Supporting Information), which illustrates the consistency between the actual structure and the theoretical optimized structure and the validity of the computational method (see Supporting Information for theoretical computational details). **Figure** **1**c shows the frontier molecular orbital diagrams of **tetra‐Pt‐dbt** monomer and dimer calculated using optimized geometries in the T_1_ excited state. For the **tetra‐Pt‐dbt** monomer, the highest occupied molecular orbitals (HOMOs) are mainly localized on the Pt atom, and the lowest unoccupied molecular orbitals (LUMOs) are delocalized throughout the whole molecule, with a significant extension to the DBS unit. This means that the monomer **tetra‐Pt‐dbt** has a mixed ^3^MLCT/^3^ILCT excited state parentage. As shown in Figure 1b, the Pt‐Pt distance of dimer [**tetra‐Pt‐dbt**]_2_ significantly decreases from 3.376 Å in the S_0_ state to 2.957 Å in the T_1_ excited state. For the monomer **tetra‐Pt‐dbt**, the Pt‐5dz^2^ orbital is HOMO‐6 (Figure 1c). After dimerization, the short Pt‐Pt distance of [**tetra‐Pt‐dbt**]_2_ results in significant energy splitting between the two Pt 5dz^2^ orbitals, lifting the 𝜎*(5dz2) orbital to the HOMO of [**tetra‐Pt‐dbt**]_2_. The LUMO of [**tetra‐Pt‐dbt**]_2_ is localized on the ligand and extends to the DBS units. Therefore, the [**tetra‐Pt‐dbt**]_2_ triplet excited state originates from a triplet metal‐metal‐to‐ligand charge transfer (^3^MMLCT) transition with low‐energy dimer emission. The calculated phosphorescence energy of dimer [**tetra‐Pt‐dbt**]_2_ is 1.90 eV, which is in good agreement with the experimentally measured phosphorescence energy of 1.87 eV. Energy decomposition analysis (EDA) was also performed on the [**tetra‐Pt‐dbt**]_2_ dimer to study the stability of the dimer aggregate. The results show that the dimer has a strong overall intermolecular interaction energy of −96.3 kcal mol^−1^ (as shown in Figure 1b), showing that [**tetra‐Pt‐dbt**]_2_ has excellent stability. A high intermolecular London dispersion interaction energy (−54.3 kcal mol^−1^) was also observed, showing strong 𝜋‐𝜋 interaction and short 𝜋‐𝜋 stacking distance in the [**tetra‐Pt‐dbt**]_2_ dimer.^[^ ^11^, ^15^ ^]^ Therefore, [**tetra‐Pt‐dbt**]_2_ dimer has good stability and is conducive to the generation of dual emission.

The square planar coordination geometry of Pt(II) complexes results in vacant axial coordination sites, which makes the complexes more susceptible to excited state structural distortion. Large structural changes will promote the electron‐vibration coupling between the excited state and the ground state, thereby strongly promoting the nonradiative decay process, resulting in a reduction of the luminescence efficiency. In order to test the role of the DBS unit, the SOC matrix element and radiative decay rate constant (*k_r_*) of **tetra‐Pt‐dbt** in the optimized S_0_ structure were calculated and compared to that of **tetra‐Pt‐dbf** (Table S1, Supporting Information). The calculated SOC matrix element and average *k_r_* value of **tetra‐Pt‐dbt** in the relaxed S_0_ structure are 38.2 cm^−1^ and 1.19 × 10^5^ s^−1^, respectively, which are higher than the corresponding values of 32.9 cm^−1^ and 8.37 × 10^4^ s^−1^ for **tetra‐Pt‐dbf**. This is consistent with our expectation that DBS can improve the SOC and radiative decay compared with DBO.

Then **tetra‐Pt‐dbt** complex was synthesized based on our previous work^[^ ^11^ ^]^ and fully characterized by ^1^H and ^13^C Nuclear Magnetic Resonance （NMR） spectroscopy, high‐resolution mass spectrometry (HRMS) and elemental analysis (see Supporting Information). We recorded the cyclic voltammogram of **tetra‐Pt‐dbt** in N, N‐dimethylformamide (DMF) with 0.1 m tetrabutylammonium hexafluorophosphate (TBAP) as the supporting electrolyte, as shown in Figure S3 (Supporting Information). **Tetra‐Pt‐dbt** shows a reversible reduction couple at −1.44 V and an irreversible oxidation wave at 1.07 V (vs [E^0^(FeCp_2_^+/0^)]). Based on the oxidation potential and reduction potential, the highest occupied molecular orbital (HOMO) and lowest unoccupied molecular orbital (LUMO) levels of **tetra‐Pt‐dbt** were estimated to be −5.11 and −2.60 eV, respectively (summarized in Table S2, Supporting Information).

**Figure** **2**a depicts the UV‐visible absorption and phosphorescence spectra of **tetra‐Pt‐dbt** in toluene solution at a concentration of 10^−5^ m, which should be attributed to **tetra‐Pt‐dbt** monomer. There are three main absorption peaks; the strong absorption wavelength ≈283 nm is assigned to intra‐ligand (IL) transition, while the relative weak absorption at 382 and 452 nm is attributed to intra‐ligand charge‐transfer (^1^ILCT) transition and metal‐to‐ligand charge‐transfer (^1^MLCT) transition, respectively.^[^ ^16^ ^]^ Green phosphorescence emission with a maximum at 529 nm was observed for **tetra‐Pt‐dbt**, with a PLQY of 0.97 under degassing conditions. The PL decay behavior of **tetra‐Pt‐dbt** in degassed toluene was also measured, showing mono‐exponential decay with a lifetime of 2.16 µs. A high radiative decay rate constant (*k_r_*) of 4.49 × 10^5^ s^−1^ was obtained, which is almost 30 times its nonradiative decay rate constant (*k_nr_*) (1.39 × 10^4^ s^−1^). For comparison, we also measured the photo‐luminescence (PL) properties of **tetra‐Pt‐dbf** under the same conditions.^[^ ^11^ ^]^ Compared with **tetra‐Pt‐dbt,** the results showed lower PLQY (0.82), longer‐decay lifetime (2.44 µs), lower *k_r_* value (3.36 × 10^5^ s^−1^) and higher *k_nr_* value (7.38 × 10 ^4^ s^−1^). These results demonstrate our molecular design that augmenting the SOC with peripheral substituents containing heavy atoms could enhance the radiative decay of Pt complexes, leading to shorter excited state lifetimes, consistent with theoretical results.

The solid‐state PL properties of **tetra‐Pt‐dbt** were also evaluated by dispersion of the complex into the wide energy gap host, 3,3′‐Di(9H‐carbazol‐9‐yl)‐1,1′‐biphenyl (mCBP). As shown in Figure 2c, significant changes in the PL spectrum were observed as the doping concentration increased from 2 to 12 wt.%. At a low concentration of 2 wt.%, the spectrum shows a maximum peak at 526 nm without a clear shoulder, consistent with the monomer emission obtained in toluene. As can be seen from Figure 2c, when [**tetra‐Pt‐dbt]** in mCBP film is 5 wt.%, the aggregate emissions are relatively low, and we estimate that the maximum concentration of **tetra‐Pt‐dbt** molecules showing only monomer emission is ≈3–4 wt.% in the film. Interestingly, as the dopant concentration increases, the emission maximum of the 12 wt.% doped film is slightly red‐shifted to 535 nm, and a gradually enhanced shoulder emission peak at 660 nm is observed. According to our previous work and the above theoretical results, this shoulder emission should be from the ^3^MMLCT excited state of the **tetra‐Pt‐dbt** aggregates. As the dopant concentration increases, aggregate formation is enhanced; therefore, the aggregates’ emission intensity gradually increases. The PLQY of these films was also measured and was 0.86, 0.93, 0.84, and 0.80 for 2, 5, 8, and 12 wt.% doped films, respectively. These values verify that high PLQY could be maintained in both monomer and aggregate states, which is beneficial to obtaining high device efficiency at different dopant concentrations.

The decay behavior of these films was also measured at the corresponding emission maxima of the main and shoulder peaks, as shown in Figure 2d and Figure S4 (Supporting Information). Table S2 (Supporting Information) summarizes excited state lifetimes of the 2, 5, 8, and 12 wt.% doped films. These lifetimes are 2.40, 2.00, 1.63, 1.19 µs at 530 nm and 2.09, 1.77, 1.64, 1.34 µs at 660 nm, respectively. As the dopant concentration increases, more aggregates are expected. This results in an increase in the MMLCT excited states and a shortened lifetime of the 660 nm emission due to its larger SOC. The increase in aggregate concentration also causes more energy transfer from the monomer to the aggregates, thereby also shortening the decay lifetime of the monomer emission observed at 530 nm. It is also worth noting the ongoing pursuit of achieving short‐lived triplet excited state lifetimes in Pt complexes, which can suppress bimolecular quenching processes (e.g., triplet‐triplet annihilation (TTA) and triplet‐polaron annihilation (TPA)). This helps reduce efficiency roll‐off and extends device operational lifetime.^[^ ^17^ ^]^

### 2.2 Device Performance

**Figure 3:**
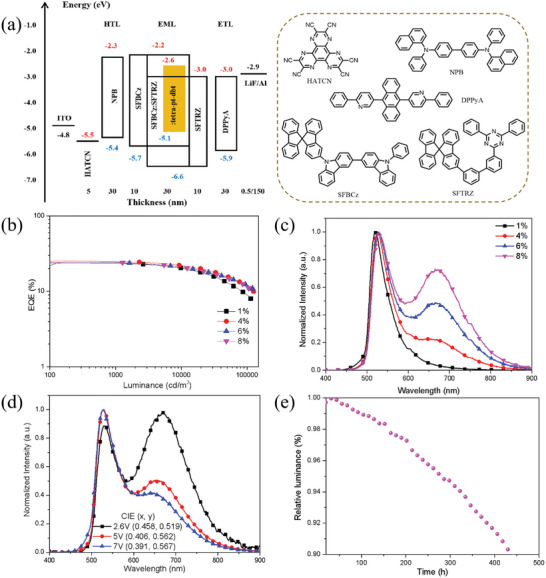
a) The structure of Device I, the energy diagram and chemical structure of the materials used in the device, b) External quantum efficiency (EQE) versus luminance characteristic of device I with different doping concentrations of **tetra‐Pt‐dbt**, c) Normalized EL spectra of Device I with different concentrations of **tetra‐Pt‐dbt** at 2.6 V, d) Normalized EL spectra of Device I with 8 wt.% **tetra‐Pt‐dbt** at 2.6, 5, and 7 V, e) Lifetimes of Device I with 8 wt.% of **tetra‐Pt‐dbt** at an initial luminance of 1000 cd m^−2^.

The electroluminescent performance of **tetra‐Pt‐dbt** as a single emitter was first evaluated using the following OLED structure (Device I): ITO/ HATCN (5nm)/ NPB (30nm)/ SFBCz (10nm)/ 50 wt.% SFBCz: 50 wt.% SFTRZ: x wt.% **tetra‐Pt‐dbt** (30nm)/ SFTRZ (10nm)/ DPPyA (30nm)/ LiF (0.5 nm)/ Al (150 nm), of which 1,4,5,8,9,11‐hexaazatriphenylene hexcarbonitrile (HATCN) and lithium fluoride (LiF) are used as hole‐ and electron‐injection layers; (4,4′‐N,N′‐bis[N‐(1‐naphthyl)‐N‐phenylamino]biphenyl) (NPB) and 9,10‐bis(6‐phenylpyridin‐3‐yl)anthracene (DPPyA) are the hole and electron transport layers; 9‐(9,9′‐spirobi[fluoren]‐3‐yl)‐9′‐phenyl‐9H,9′H‐3,3′‐bicarbazole (SFBCz) and 2‐(3′‐(9,9′‐spirobi[fluoren]‐3‐yl)‐[1,1′‐biphenyl]‐3‐yl)‐4,6‐diphenyl‐1,3,5‐triazine (SFTRZ) are electron and hole blocking layers, respectively. Previously reported stable SFBCz: SFTRZ was chosen as the exciplex‐forming host,^[^ ^18^ ^]^ and doping concentration of **tetra‐Pt‐dbt** varied from 1, 4, 6, to 8 wt.%, respectively. The energy level diagram and chemical structures of the materials used in the devices are shown in **Figure** **3**a. We also fabricated **tetra‐Pt‐dbf** based OLEDs with the same device structures for comparison, and their performance data are provided in Figure S5 and Table S3 (Supporting Information).

Figure 3c provides the electroluminescence spectrum of the device recorded at 1000 cd m^−2^. Compared with the PL results, the same trend is observed with increasing dopant concentration, which is the intensity of shoulder emission at 660 nm gradually increases while the main peak maximum is in the range of 520–530 nm. This shows that both monomer emission and aggregates emission can be maintained under electrical excitation. We also studied the variation of EL spectra with increasing operating voltage, as shown in Figure 3d for the 8 wt.% doped device and Figure S7 (Supporting Information). For the device with a doping concentration of 1 wt.%, only one emission peak is observed at 520 nm, which remains essentially unchanged when the voltage increases from 2.6 to 7.0 V; the CIE coordinates at 2.6 and 7.0 V are (0.292, 0.636) and (0.283, 0.607), respectively. Since no shoulder emission peak is observed, only monomer emission exists for this device and therefore no color‐tunable properties are observed.

In contrast, clear voltage‐dependent color‐tunable characters were recorded for the other three devices with both monomer emission and aggregates emission (Device I with 4 wt.%, 6 wt.% and 8 wt.%). When the operating voltage increases from 2.6 to 7.0 V, the normalized EL spectra of the three devices show a gradually enhanced monomer emission peaked at ≈530 nm and a relatively low ratio of aggregation emission peaked at around 660 nm (Depicted in Figure 3d). This is consistent with our previous results obtained from **tetra‐Pt‐dbf**, and the reason for the voltage‐dependent emission color should be attributed to the competition between charge‐trapping and direct energy transfer mechanisms at different operation voltages.^[^ ^12^, ^19^ ^]^ Since the excitation energy of the aggregates is low, it is believed that charges can easily recombine on aggregates at low operation voltage, resulting in a relatively high aggregates emission intensity. As the voltage increases, more charges will recombine on the monomer, thereby gradually increasing the emission intensity of the monomer. Due to the strong intermolecular bonding energy of **tetra‐Pt‐dbt**, the 4 wt.% doped device exhibits obvious voltage‐dependent color changes. As the dopant concentration increases, a wider range of color changes can be observed. For the 8 wt.% doped device, the aggregates emission outperforms the monomer emission even at low operation voltages due to the higher aggregate concentration and the CIE coordinates from (0.458, 0.519) at 2.6 V to (0.391, 0.567) at 7 V (Figure 3d) has a large shift.

Figure 3b depicts the EQE‐luminance characteristics of these devices and is also summarized in Table S3 (Supporting Information). Interestingly, due to their similar PLQY, all four devices also exhibit similar EQEs ranging from 23.9% to 25.5%, with the 4 wt.% doped device showing the highest maximum EQE. Additionally, these devices exhibit fairly small efficiency roll‐off, with all EQE drops <3% at high luminance of 1000 cd m^−2^. In addition to employing exciplex‐forming hosts, it is believed that short‐lived excited states also contribute to this small EQE roll‐off.^[^ ^20^ ^]^ It also worth noting that devices based on the reference emitter (**tetra‐Pt‐dbf**) exhibit inferior EQEs in the range of 20.9–23.6%, consistent with the relatively lower PLQYs of **tetra‐Pt‐dbf**. These results validate the superiority of our newly designed platinum emitter, which can maintain high emission efficiency in both the monomer and aggregate states. The operational stability of these devices was also measured at a constant current density with an initial luminance of 1000 cd m^−2^. As can be seen from Figure 3e, the lifetime at 90% of the initial luminance (LT_90_) is 440 h. By adopting a degradation acceleration factor that links luminance to lifetime, the LT_90_ at an initial luminance of 100 cd m^−2^ can be extrapolated by using the equation, LT_90_ (100 cd m^−2^) = LT_90_ (1000 cd m^−2^) × (1000 cd m^−2^/100 cd m^−2^) ^n^, with n = 1.75,^[^ ^11^, ^21^ ^]^ the estimated LT_90_ at 100 cd m^−2^ luminance is 24743 h.

**Figure 4:**
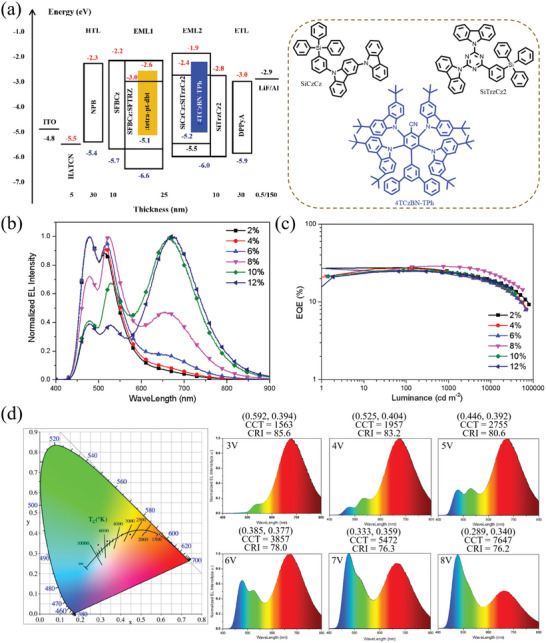
a) The structure of Device II, the energy diagram and chemical structure of the materials used in EML2, b) Normalized EL spectra of WOLEDs recorded at 35 mA cm^−2^ using different concentrations of **tetra‐Pt‐dbt**, c) EQE versus luminance characteristic of WOLEDs, d) CIE coordinates and emission spectra of W6 recorded at 3, 4, 5, 6, 7, and 8 V.

Based on the excellent voltage‐dependent performance of **tetra‐Pt‐dbt,** we designed an advanced double‐emissive‐layer hybrid WOLEDs (Device II) with the structure of ITO/ HATCN (5 nm)/ NPB (30 nm)/ SFBCz (10 nm)/ EML1 (10 nm)/ EML2 (15 nm)/ SiTrzCz2 (10 nm)/ DPPyA (30 nm)/ LiF (0.5 nm)/ Al (150 nm) (**Figure** **4**a). Here, emissive layer (EML)1 is composed of 50 wt.% SFBCz, 50 wt.% SFTRZ, and 13 wt.% **tetra‐Pt‐dbt**, which provides green emission and red emission. EML2, composed of 65% 9‐(3‐(triphenylsilyl)phenyl)‐9H‐3,9′‐bicarbazole (SiCzCz), 35% 9,9′‐(6‐(3‐(triphenylsilyl)phenyl)‐1,3,5‐triazine‐2,4‐diyl)bis(9H‐carbazole) (SiTrzCz2) and 30% 2,3,5,6‐tetrakis(3,6‐di‐tertbutyl‐9H‐carbazol‐9‐yl)‐5′‐phenyl‐[1,1′:3′,1′'‐terphenyl]‐4‐carbonitrile (4TCzBN‐TPh), could provide blue emission. SiCzCz: SiTrzCz2 is a reported stable exciplex‐forming host for blue emitter,^[^ ^22^ ^]^ while 4TCzBN‐TPh is an efficient, stable blue TADF emitter. Since the blue emission component is often an important determining part of device stability, EML2 is expected to provide stable blue emission. The concentration optimization range of **tetra‐Pt‐dbt** is 2, 4, 6, 8, 10, and 12 wt.%, corresponding to W1, W2, W3, W4, W5 and W6. All results are provided in Figure 4 and **Table** **1**.

**Table 1:** Key performance characteristics of WOLEDs.

Device No.	Turn‐on ^a)^ [V]	L^b)^ [cd/m^2^]	EQE [%]		CE_max_ ^c)^ [cd A^−1^]	PE_max_ ^d)^ [lm W^−1^]	CIE (x,y)		CCT [K]		CRI_max._
			Max.	at 1000 cd m^−2^			At 2.6 V	At 8.0 V	At 2.6 V	At 8.0 V	
W1	2.5	79 380	27.6	23.7	67.1	81.0	0.343, 0.567	0.181, 0.324	5354	22 870	47.2
W2	2.5	69 620	27.0	23.4	65.2	73.2	0.363, 0.561	0.188, 0.330	5012	19 490	60.7
W3	2.5	66 380	25.2	22.3	49.8	53.1	0.413, 0.529	0.194, 0.330	4130	18 040	81.1
W4	2.4	56 530	28.8	28.6	46.3	42.2	0.502, 0.474	0.211, 0.334	2628	14 860	81.5
W5	2.4	38 280	25.5	24.4	24.6	22.4	0.549, 0.434	0.247, 0.330	1950	11 010	86.4
W6	2.4	29 640	24.7	23.7	18.1	15.8	0.613, 0.378	0.289, 0.340	1563	7647	85.6

^a)^ Voltage at the luminance of 1.0 cd m^−2^;

^b)^ Luminance at 8.0 V;

^c)^ Maximum current efficiency;

^d)^ Maximum power efficiency.

Figure 4b provides the normalized electroluminescence spectrum of CT‐WOLED recorded at 1000 cd m^−2^, which shows three emission peaks at 478 nm from 4TCzBN‐TPh and 529 / 662 nm from **tetra‐Pt‐dbt** monomer and aggregates. Benefiting from the advantages of **tetra‐Pt‐dbt** dual emission, two emitters could realize tri‐color WOLEDs, simplifying the device structure. It is also worth noting that as the concentration of **tetra‐Pt‐dbt** increases, a gradual decrease in the intensity of blue light component of 4TCzBN‐TPh is observed, indicating that more excitons are located in the **tetra‐Pt‐dbt** monomer and aggregates. Figure 4c depicts the EQE‐luminance characteristics of these devices, all showing high maximum EQEs in the range of 25.5–28.8%. This is due to the high luminescent efficiency of 4TCzBN‐TPh as a blue emitter and **tetra‐Pt‐dbt (**monomer/aggregate) as complementary color emitter. In particular, the highest EQE_max_ of 28.8% was achieved for the 8 wt.% **tetra‐Pt‐dbt** doped device at the luminance of 378 cd m^−2^, representing one of the most efficient CT‐WOLEDs. All these devices exhibit a small efficiency roll‐off, with EQE remaining in the range of 22.3–28.6% at 1000 cd m^−2^. Especially, the 8 wt.% **tetra‐Pt‐dbt** doped device shows an EQE of 26.2% even at a luminance of 5000 cd m^−2^. It was found that the 2 wt.% **tetra‐Pt‐dbt** doped device achieved the highest maximum power efficiency of 81.0 lm W^−1^.

The color tunability of these devices was analyzed as the operation voltage increased from 3 to 8 V, and the changes in spectra, CIE coordinates and correlated color temperature (CCT) are provided in Figure 4d, Figures S8–S12 (Supporting Information) and Table 1. All these devices exhibit large color changes with increasing voltage, emitting dull warm white light at low voltages and bright cool emission at high voltages. As shown in Figure 4d, at low operating voltage, the aggregates emission of **tetra‐Pt‐dbt** becomes the main part due to its low energy. As the driving voltage increases, the intensity of blue emission and green emission gradually increases. The change in the color emission originates not only from the voltage dependent monomer emission and aggregates emission of **tetra‐Pt‐dbt**, but also from the exciton recombination zone shifted from EML1 to EML2 with increasing voltage, which enhances 4TCzBN‐TPh blue emission component. In particular, for the 12 wt.% doped device, its CIE and CCT change from (0.613, 0.378) to (0.289, 0.340) and 1563 to 7647, respectively, with a maximum color rendering index (CRI) of 85.6. These changes agree well with the blackbody radiation curve, showing that the device has good color quality as CT‐WOLED and can well meet different daily scenarios from a circadian rhythm perspective; that is, bright cool light with high CCT can improve people activity and alertness during daytime, and dim warm light with low CCT at night can maintain human health.

**Figure 5:**
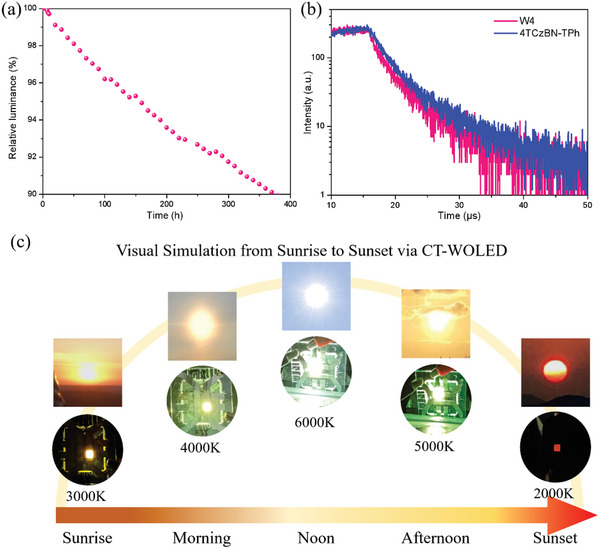
a) Lifetime of Device II with 8 wt.% **tetra‐Pt‐dbt** doped device (W4) at an initial luminance of 1000 cd m^−2^, b) EL decay curves of W4 and the monochromic device using 65% SiCzCz, 35% SiTrzCz2 and 30% 4TCzBN‐TPh as single emissive layer measured at 478 nm, c) Visual emulation of sunlight from sunrise to noon to sunset using CT‐WOLED (color temperature: 2000 to 6000 K).

Due to its highest efficiency, we further evaluate the operational lifetime of OLEDs (W4) with 8 wt.% **tetra‐Pt‐dbt** at a constant current density and an initial brightness of 1000 cd m^−2^. As shown in **Figure** **5**a, an impressive long LT_90_ of 376 h was observed. By using a degradation acceleration factor that links luminance to lifetime, the LT_90_ for an initial luminance of 100 cd m^−2^ can be extrapolated to 21144 h using the equation of LT_90_ (100 cd m^−2^) = LT_90_ (1000 cd m^−2^) × (1000 cd m^−2^/100 cd m^−2^)^n^, with n = 1.75.^[^ ^11^, ^21^ ^]^ To the best of our knowledge, this is the longest device lifetime among CT‐WOLEDs in the literature (summarized in Table S4, Supporting Information). The good operational stability of the device should naturally benefit from the good stability of 4TCzBN‐TPh and **tetra‐Pt‐dbt**. In addition, we also measured the electroluminescence curve of 4TCzBN‐TPh in this WOLED, as shown in Figure 5b, and measured the decay curve of the blue device using 4TCzBN‐TPh directly as the emitter for comparison. Clearly, 4TCzBN‐TPh in this WOLED shows a shorter exciton lifetime compared to the curves obtained from the blue device. This may be attributed to the energy transfer from high‐energy 4TCzBN‐TPh excited state to the low‐energy **tetra‐Pt‐dbt** (monomer and aggregate) excited states. As we all know, the stability of the blue light emitting component is the bottleneck of the entire white device. The acceleration of exciton consumption in 4TCzBN‐TPh could in turn enhance the stability of the blue light emitting part, ultimately contributing to the significant long lifetime of WOLED.

Given that our CT‐WOLED has good color‐temperature tunability and decent device stability, we initially explored its potential applications in the field of visual sunlight emulation. As shown in Figure 4d, our CT‐WOLED can continuously emit white light with different color temperatures from 2000 to 6000 K. Therefore, we adopt this CT‐WOLED to visually simulate the progression of sunshine from sunrise (3000 K) to noon (6000 K) to sunset (2000 K). The emitted light of CT‐WOLED is highly consistent with sunlight at different times throughout the day, as shown in Figure 5c. We believe this device has the potential to be used for dynamic sunlight emulation or day‐night cycle emulation in the virtual reality (VR) field. For instance, such CT‐WOLED, when integrated with a smart drive system, is capable of visually emulating the natural progression of sunlight from sunrise to sunset, thereby assisting patients with mobility impairments or postpartum women in regulating their circadian rhythms, which potentially facilitate their recovery process; in addition, considering the characteristics of the flat light source and the simplicity of the drive requirements, our unique CT‐WOLED is more suitable for dynamic sunlight emulation than traditional multi‐LED systems or systems composed of multiple monochromatic OLEDs.^[^ ^2^, ^23^ ^]^

### 2.3 General Remarks

#### 2.3.1 Discussion on Voltage‐Dependent Color‐Tunable Mechanism

Our previous work conducted extensive studies on voltage‐dependent color‐tunable emission.^[^ ^11^, ^12^ ^]^ In order to better explain the mechanism of this excellent color tunability performance, we adopt the classic “trapping and energy‐transfer” model to simulate the experimental data; this model can be expressed by Equation (1).

(1) $$q\;\left(U\right)=\frac{D}{\mu }\;{\left(\frac{d}{LT}\right)}^{2}\frac{1}{U-U_{0}}+\frac{d}{LT}$$

D is the diffusion coefficient of trapped electrons, μ is the carrier mobility, d is the EML thickness, LT is the average diffusion distance before the carrier reaches the trapping center, U is the driving voltage, and U_0_ is the built‐in electronic field. D/μ is the Einstein relation describing the ratio between diffusivity and mobility. Equation (1) was derived by Meerholz and coworkers^[^ ^19^ ^]^ for single‐layer polymer OLEDs and applied to multilayer OLEDs by Wang and coworkers^[^ ^24^ ^]^ to explain CT‐OLEDs. By comparing the experimental data with the “trapping and energy‐transfer” model, we believe that this color‐tunability phenomenon of CT‐WOLED should be mainly due to the competition between charge trapping and direct energy transfer to the **tetra‐Pt‐dbt** emitter.

In OLED devices, the charge mobility of the transport layer material is affected by voltage. Changes in voltage will lead to changes in the injection and transport behavior of carriers (electrons/holes), affecting charge mobility. Specifically, voltage changes affect the injection efficiency, transport speed, and distribution of carriers within the transport layer, which in turn affects charge mobility. For example, Figure 2e of our previous work^[^ ^25^ ^]^ shows that the charge mobility of DPPyA film depends on the voltage.

#### 2.3.2 Effect of SOC on Radiative/Nonradiative Decay

Due to the different spin multiplicities of triplet and singlet states, the T_1_→S_0_ transition is spin‐forbidden. Large spin‐orbit coupling (SOC) matrix element can relax spin‐forbidden electronic transition and increase the radiative decay rate. Furthermore, in order to achieve a high‐efficiency ISC process, the d‐orbitals of the singlet excited state and the emitting T_1_ excited state need to have different orientations.^[^ ^26^ ^]^ The non‐radiative decay rate, *k_nr_*, is also related to SOC. Meanwhile, the larger SOC matrix elements will also affect the nonradiative decay process and leads to a larger *k_nr_* value. However, when comparing the results of **tetra‐Pt‐dbt** (*k_nr_* 1.39 × 10^4^ s^−1^; SOC 38.2 cm^−1^) and **tetra‐Pt‐dbf** (*k_nr_* 7.38 × 10 ^4^ s^−1^; SOC 32.9 cm^−1^), the SOC matrix element of **tetra‐Pt‐dbt** is a little larger, but the corresponding k_nr_ of **tetra‐Pt‐dbt** is much lower. We speculate that this may be due to the relatively small impact of the SOC matrix element on *k_nr_*, while the degree of structural distortion when transitioning from the T_1_ state to the S_0_ state has a more significant impact on *k_nr_*.^[^ ^26c^ ^]^ The previously calculated SOC matrix elements and *k_r_* (s) for **tetra‐Pt‐dbt** and **tetra‐Pt‐dbf** are listed in **Table** **2**.

**Table 2:** List of SOC matrix elements and *k_r_* for complexes **tetra‐Pt‐dbt** and **tetra‐Pt‐dbf**.

Complexes	SOC matrix elements [cm^−1^]	*k_r_* [s^−1^]
**tetra‐Pt‐dbt**	38.2	1.19 × 10^5^
**tetra‐Pt‐dbf**	32.9	8.37 × 10^4^

#### 2.3.3 Discussion on PLQYs of tetra‐Pt‐dbt and Relevant Device Efficiency

We find that as the doping concentration increases, the PLQY of **tetra‐Pt‐dbt** first increases and then decreases. At low concentrations, the distance between molecules of the luminescent materials is large, allowing the luminescent molecules to emit light freely. However, as concentration increases, the distance between molecules decreases, leading to an increase in nonradiative energy transfer between molecules and a decrease in fluorescence efficiency. In aggregated or solid‐state forms, the planar conjugated structure of **tetra‐Pt‐dbt** molecules is prone to π–π stacking, further exacerbating fluorescence quenching. The **tetra‐Pt‐dbt** in mCBP from 2 to 5 wt.% may not yet trigger the concentration quenching effect, resulting in a slight increase in PLQY. However, further increasing concentration results in significant concentration quenching, which originates from the triplet‐related quenching.

EQE is related to parameters such as charge injection efficiency, spin factor, PLQY, and light out‐coupling efficiency. Different concentrations of **tetra‐Pt‐dbt** may lead to variations in these parameters, which in turn lead to the change of EQE.

Generally speaking, the EQE value is mainly determined by the PLQY of an emitter and the out‐coupling efficiency of the device; the horizontal transition dipole moment could significantly enhance the out‐coupling efficiency. Since the aggregates of planar Pt (II) molecules prefer to form horizontal emitting dipole orientation,^[^ ^27^ ^]^ we thereby infer that in the EML of Device I with a doping concentration of 8 wt.%, some aggregates were formed. The aggregates formed under this condition have a higher horizontal dipole ratio, thereby improving the out‐coupling efficiency and the EQE_max_ of the device. Thus, Device I containing 8 wt.% **tetra‐Pt‐dbt** showed a higher EQE_max_.

## 3 Conclusion

In this work, we present an advanced design of high efficiency, long lifetime tri‐color hybrid CT‐WOLEDs based on hybrid blue TADF and voltage‐dependent green‐to‐red phosphorescence. The key is that a single Pt complex can achieve color‐tunable monomer and aggregate emission with high efficiency and good stability. This design draws on our previous work to employ DBS units with sulfur atoms to enhance the SOC to achieve fast radiative decay and high PLQY. The proof‐of‐the‐concept emitter **tetra‐Pt‐dbt** exhibits green emission at 529 nm and red emission at 662 nm in a device with an EQE of over 25% and a long LT_90_ of 24743 h at the 100 cd m^−2^ luminance; and CIE range of this device is wide, from (0.511, 0.471) at 2.6 V to (0.441, 0.536) at 8V. The simplified double‐emissive‐layer WOLEDs inherit a wide color‐tunable range with CIE coordinates and CCT shift from (0.502, 0.474) and 2628 K at 2.6 V to (0.211, 0.334) and 14860 K at 8 V, respectively. In addition, not only a high EQE of 28.8% at 378 cd m^−2^ to 26.2% at 5000 cd m^−2^ is obtained, but also an impressively long LT_90_ of >21 144 h at 100 cd m^−2^ luminance, representing one of the most stable and efficient CT‐WOLEDs. Our work provides an effective design strategy to build a tri‐color CT‐WOLED with high efficiency, ultralong operational lifetime, wide color‐tunability range, and simple device structure, paving the way for the commercialization of healthy solid‐state lighting. Owing to the high consistency with the full‐day sunshine lighting effects via sunrise‐sunset visual simulation, our cutting‐edge CT‐WOLEDs are poised to facilitate the applications in the regulation of circadian rhythms in healthcare, dynamic visual‐sunshine in VR, and beyond.

## Conflict of Interest

The authors declare no conflict of interest.

## Supporting information



Supporting Information

## Data Availability

The data that support the findings of this study are available on request from the corresponding author. The data are not publicly available due to privacy or ethical restrictions.
