# Supplementary material for: High‐Efficiency, Long‐Lifetime and Color‐Tunable Hybrid WOLEDs Using a Platinum Complex with Voltage‐Dependent Monomer and Aggregate Emission
Source: Adv Sci (Weinh). 2025 Jan 21;12(13):2411364. doi: 10.1002/advs.202411364 (PMC11967765; doi:10.1002/advs.202411364)
Supplement: Supplementary file 1 — Supporting Information [file ADVS-12-2411364-s001.docx]

Supporting Information

High-efficiency, Long-lifetime and Color-tunable Hybrid WOLEDs Using a Platinum Complex with Voltage-dependent Monomer and Aggregate Emission

*Yangyang Xin, Mao Mao*, Shuo Xu, Kaixin Tan, Gang Cheng, Hai Zhang, Hengyi Dai, Tianyu Huang, Dongdong Zhang*, Lian Duan*, and Chi-Ming Che**

**1. General Procedures**

**1.1 General Information.**

All commercially available reagents were used as received unless otherwise stated. All reactions were carried out using Schlenk techniques under a nitrogen atmosphere. NMR spectra were measured on a Bruker 400/600 MHz spectrometer with the internal standard of tetramethylsilane (TMS). Mass spectra were recorded on a Shimadzu MALDI-TOF mass spectrometer.

**1.2 Electrochemical measurement.**

The electrochemical measurements were performed with a Potentiostat/Galvanostat Model 283 (Princeton Applied Research) electrochemical workstation by using Pt as the working electrode, platinum wire as the auxiliary electrode, and an Ag wire as the reference electrode standardized against ferrocene/ferrocenium. The oxidation potentials were measured in dichloromethane (CH2Cl2) solution containing 0.1 M n-Bu4NPF6 as the supporting electrolyte at a scan rate of 100 mV s^-1^. The reduction potentials were measured in N, N-Dimethylformamide (DMF) solution containing 0.1 M n-Bu4NClO4 as the supporting electrolyte at a scan rate of 100 mV s^-1^.

**1.3 Photophysical characterization.**

Organic films for optical measurements were fabricated by thermal evaporation under high vacuum onto clean quartz substrates. UV-vis absorption spectra were recorded by an Agilent 8453 spectrophotometer. Fluorescence and phosphorescence spectra at steady state were recorded by Hitachi F-7000 Fluorescence Spectrometer. Fluorescence lifetime measurement was carried out with Edinburgh fluorescence spectrometer (FLS1000) using picosecond pulsed diode laser under the excitation at 375 nm. Photoluminescence quantum yields were measured by Hamamatsu absolute PL quantum yield spectrometer (C9920-02G) with an integrating sphere.

**1.4 Device Fabrication and Characterization.**

Before device fabrication, the ITO glass substrates were precleared carefully. Then the sample was transferred to the deposition system. The devices were prepared in vacuum at a pressure of 5 × 10^-6^ Torr. The organic layers were thermally evaporated at a rate of 1.0 Å s^-1^. After the organic film deposition, 0.5 nm of LiF and 150 nm of aluminum were thermally evaporated onto the organic surface. All of the organic materials used were purified by a vacuum sublimation approach. The electrical characteristics of the devices were measured with a Keithley 2400 source meter. The electroluminescence spectra and luminance of the devices were obtained on Hamamatsu external quantum efficiency measurement system. All the device fabrication and characterization steps were carried out at room temperature under ambient laboratory conditions.

**1.5 Theoretical computational details.**

The transition dipole moments, oscillator strength, spin-orbit coupling (SOC) constant, and radiative rate constant for T_1_→S_0_ transitions were calculated by the perturbative SOC TDDFT (pSOC-TDDFT) method^1^ in the ADF2019 package^2-3^. The calculations were performed at the level of ZORA (Zeroth Order Regular Approximation)^4-6^-M06^7^/TZP^8-9^ using the optimized ground state geometries. Solvent effects were considered by means of the Conductor like Screening Model (COSMO) with toluene as the solvent^10-11^.

DFT/TDDFT calculations were performed at the level of hybrid functional M06^7^ with D3 dispersion correction^12^ using Gaussian 16 program package. The 6-31G*^13-14^ basis set was employed for all atoms except platinum, which was described by the LANL2DZ basis set and corresponding effective core potentials (ECPs) proposed by Hay and Wadt.^15-16^ Solvent effects were taken into account using the polarizable continuum model (PCM) with toluene as solvent.^17^

The energy decomposition analysis (EDA) was performed with the Amsterdam Density Functional 2019 package.^18^ All electron Slater-type orbital (STO) basis set of TZP (valence triple-ζ polarization function) was applied for all elements.^8-9^ Zeroth Order Regular Approximation (ZORA) was employed for scalar relativistic effects.^19-21^ Solvent effects were considered by means of the Conductor like Screening Model (COSMO) with toluene as the solvent.^10^ EDA decomposes total intermolecular interactions into London dispersion, orbital interaction, Pauli repulsion, and electrostatic interaction (eq. 1)

$$E_{int}=E_{Disp}+E_{Orb}{+E_{Pauli}+E}_{Elst} (1)$$

**2. Synthesis and properties of tetra-Pt-dbt**

**Scheme S1.** Synthetic procedures of tetra-Pt-dbt:

**Synthesis of compound 2:** Compound **1** (57.6 g, 100 mmol), 2,4-dichloro-6-methylpyrimidine (48.6 g, 300 mmol) and sodium hydroxide (12.0 g, 300 mmol) were dissolved in 1,4-dioxane (600 mL) and water (150 mL). The mixture was degassed under Argon bubbling for 5 minutes and then Tetrakis(triphenylphosphine)palladium(0) (1.16 g, 1 mmol) was added. The mixture was heated to 80 °C for 2 h under Argon and then cooled to room temperature. After phase separation, the organic phase was collected and the aqueous phase was extracted with dichloromethane (3 x 100 mL). The combined organic phase was washed with brine (3 x 300 mL) and vaporized under reduced pressure. The product was purified through a silica gel column (800 g, eluent: hexane/ethyl acetate (v/v) = 50/1 to 10/1) and collected as a white solid (isolated yield: 99.3% (57.1 g)). ^1^H NMR (400 MHz, CDCl_3_) δ 8.72 (t, J = 1.8 Hz, 1H), 8.26 (dt, J = 7.8, 1.4 Hz, 1H), 8.10 – 8.03 (m, 3H), 7.95 (d, J = 1.5 Hz, 1H), 7.69 – 7.54 (m, 5H), 7.44 (ddd, J = 8.3, 7.3, 1.8 Hz, 1H), 7.22 – 7.13 (m, 2H), 7.08 (dd, J = 8.3, 1.1 Hz, 1H), 3.94 (s, 3H), 2.64 (s, 3H), 1.44 (s, 18H).

**Synthesis of compound 3:** Compound **2** (57.5 g, 100 mmol), 4-dibenzothiopheneboronic acid (34.2 g, 150 mmol), potassium phosphate (63.7 g, 300 mmol) and Sphos (1.64 g, 4 mmol) were dissolved in tetrahydrofuran (800 mL) and water (400 mL). The mixture was degassed under Argon bubbling for 5 minutes and then palladium acetate (449 mg, 2 mmol) was added. The mixture was heated to 70 °C for 2 h under Argon and then cooled to room temperature. After phase separation, the organic phase was collected and the aqueous phase was extracted with dichloromethane (3 x 100 mL). The combined organic phase was washed with brine (3 x 300 mL) and vaporized under reduced pressure. The product was purified through a silica gel column (800 g, eluent: hexane/ethyl acetate (v/v) = 30/1 to 7/1) and collected as a pale-yellow oil (isolated yield: 96.1% (69.5 g)). ^1^H NMR (400 MHz, CDCl_3_) δ 8.83 (t, J = 1.8 Hz, 1H), 8.30 – 8.14 (m, 4H), 8.12 – 8.05 (m, 3H), 7.98 (d, J = 1.5 Hz, 1H), 7.83 – 7.78 (m, 1H), 7.69 – 7.54 (m, 6H), 7.53 – 7.44 (m, 3H), 7.39 (ddd, J = 8.3, 7.4, 1.8 Hz, 1H), 7.12 – 7.02 (m, 2H), 3.91 (s, 3H), 2.78 (s, 3H), 1.42 (s, 18H).

**Synthesis of compound 4:** Compound 3 (14.4 g, 20 mmol) and pyridine hydrochloride (115.6 g, 1 mol) were roughly stirred and grinded with a glass rod. Then the mixture was heated to 160 °C for 12 h under vigorous stir. After cooling down, potassium carbonate solution (2 M, 600 mL) and dichloromethane (300 mL) were added. The aqueous phase was extracted with dichloromethane (2 x 100 mL) and the combined organic phase was vaporized under reduced pressure after drying over magnesium sulphate. The product was purified through a silica gel column (330 g, eluent: hexane/ethyl acetate (v/v) = 20/1 to 5/1) and collected as a bright yellow solid (isolated yield: 87.7% (12.4g)). ^1^H NMR (400 MHz, CDCl_3_) δ 8.73 (t, J = 1.8 Hz, 1H), 8.26 – 8.17 (m, 3H), 8.13 (dt, J = 7.9, 1.4 Hz, 1H), 8.07 (dd, J = 7.1, 1.4 Hz, 2H), 8.01 – 7.95 (m, 2H), 7.84 – 7.78 (m, 1H), 7.70 (t, J = 7.7 Hz, 1H), 7.66 – 7.57 (m, 5H), 7.54 (d, J = 1.4 Hz, 1H), 7.48 (ddd, J = 7.0, 4.9, 1.6 Hz, 2H), 7.37 (ddd, J = 8.4, 7.1, 1.6 Hz, 1H), 7.09 (dd, J = 8.2, 1.2 Hz, 1H), 6.99 (ddd, J = 8.1, 7.1, 1.3 Hz, 1H), 2.79 (s, 3H), 1.45 (s, 18H).

**Synthesis of compound 5 (tetra-Pt-dbt):** Compound 4 (10.6 g, 15 mmol) was dissolved in chloroform (150 mL) and then Potassium tetrachloroplatinate(II) (8.3 g, 20 mmol, grinded), tetrabutylammonium bromide (3.2 g, 10 mmol), acetic acid (1.5 L) and water (10 mL) was added. The mixture was heated to reflux for 12 h under Argon and then cooled to room temperature. The solvent was removed under reduced pressure and the solid residue was dissolved in dichloromethane (500 mL). The organic phase was washed with water (3 x 500 mL) and brine (2 x 300 mL), then the organic phase was dried over magnesium sulphate and vaporized under reduced pressure. The product was isolated through a silica gel column (330 g, eluent: hexane/dichloromethane/acetone (v/v/v) = 80/20/1 to 80/20/5), then, further purified by recrystallization in dichloromethane/methanol (100/800 mL) and collected as an orange solid (isolated yield: 79.5% (10.8 g)). ^1^H NMR (400 MHz, CD_2_Cl_2_) δ 8.16 (d, J = 1.8 Hz, 1H), 8.03 (d, J = 2.1 Hz, 1H), 7.99 – 7.88 (m, 3H), 7.69 – 7.54 (m, 7H), 7.54 – 7.45 (m, 3H), 7.41 – 7.34 (m, 2H), 7.28 (ddd, J = 8.3, 6.6, 1.7 Hz, 1H), 7.21 (t, J = 7.6 Hz, 1H), 7.15 (dd, J = 8.4, 1.4 Hz, 1H), 6.55 (ddd, J = 8.2, 6.6, 1.4 Hz, 1H), 3.17 (s, 3H), 1.43 (s, 18H). ^13^C NMR (400 MHz, CD_2_Cl_2_) δ 171.37, 168.25, 165.37, 164.99, 164.86, 164.55, 160.96, 156.10, 153.55, 152.57, 152.00, 149.43, 145.50, 142.11, 141.28, 138.24, 130.85, 129.56, 127.73, 126.38, 125.45, 125.43, 123.68, 123.64, 123.37, 123.35, 123.22, 123.11, 122.76, 122.14, 121.66, 121.52, 121.47, 120.80, 120.26, 118.56, 115.38, 114.13, 113.96, 111.88, 35.07, 31.34, 24.78.


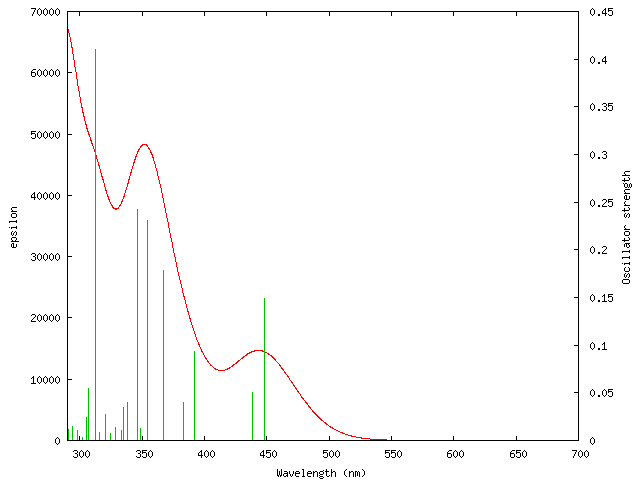


**Figure S1.** The simulated absorption spectrum of **tetra-Pt-dbt** in the optimized ground state geometry in toluene solution.

**Table S1.** The calculated SOC coupling constant and k_r_ values of **tetra-Pt-dbt** and **tetra-Pt-dbf**.

|  | S_0_ structure | | | | |
| --- | --- | --- | --- | --- | --- |
|  | T_1_^0^ | T_1_^1^ | | | T_1_^2^ |
| **tetra-Pt-dbt** |  |  | | |  |
| *μ*T1 S0 | 0.044 | 0.071 | | | 0.11 |
| *f* | 0.00074 | 0.000022 | | | 0.0006 |
| ∆E_T1-S0_ | 2.46 | 2.47 | | | 2.47 |
| $\vert\left\langle\psi_{S1}\vert{\hat{\mathcal{H}}}_{\mathrm{SO}}\vert\psi_{T1} \right\rangle\vert$ | 38.2 | | | | |
| *k*_r_ | 1.94 × 10^5^ | 5.79 × 10^3^ | | | 1.59 × 10^5^ |
| *k*_r,avg_ | 1.19 × 10^5^ | | | | |
| **tetra-Pt-dbf** |  | | | | |
| *μ*T1 S0 | 0.045 | 0.13 | | | 0.13 |
| *f* | 0.00052 | 0.00010 | | 0.00035 | |
| ∆E_T1-S0_ | 2.45 | 2.45 | | 2.45 | |
| $\vert\left\langle\psi_{S1}\vert{\hat{\mathcal{H}}}_{\mathrm{SO}}\vert\psi_{T1} \right\rangle\vert$ | 32.9 | | | | |
| *k*_r_ | 1.33 × 10^5^ | 2.63 × 10^4^ | 9.18 × 10^4^ | | |
| *k*_r,avg_ | 8.37 × 10^4^ | | | | |

*μ*T1 S0 is transition dipole moment (Debye), *f* is oscillator strength, $|\left\langle\psi_{S1}|{\hat{\mathcal{H}}}_{\mathrm{SO}}|\psi_{T1} \right\rangle|$ is SOC matrix element, *k_r_* is radiative decay rate constant (s^-1^) for T_1_ → S_0_ transition.

**Table S2.** Selected photophysical data of **tetra-Pt-dbt**.

|  | Medium | *λ_abs_* [nm]  (*ε* ×10^4^ [M^-1^ cm^-1^]) | λ_em_ [nm] | Φ_em_ | τ_em_ [μs] | *k*_r_ [10^5^ s^-1^] | E_T_ [eV] | HOMO^a^ (eV) | LUMO^a^ (eV) |
| --- | --- | --- | --- | --- | --- | --- | --- | --- | --- |
| **tetra-Pt-dbt** | Toluene  (298 K) | 283 (6.17),  382 (1.34),  448 (0.61) | 529 | 0.97 | 2.16 | 4.49 | 2.52 | -5.11 | -2.60 |
|  | 2 wt% mCBP (298 K) | / | 525 | 0.86 | 2.40,  2.09 |  |  |  |  |
|  | 5 wt% mCBP (298 K) | / | 529, 655 | 0.93 | 2.00,  1.77 |  |  |  |  |
|  | 8 wt% mCBP (298 K) | / | 534, 662 | 0.84 | 1.63,  1.64 |  |  |  |  |
|  | 12 wt% mCBP (298 K) | / | 535, 665 | 0.80 | 1.19,  1.34 |  |  |  |  |

^a^ estimated peak potential from CV measurements in DMF using Cp_2_Fe^0/+^ values of 4.8 eV below the vacuum level.


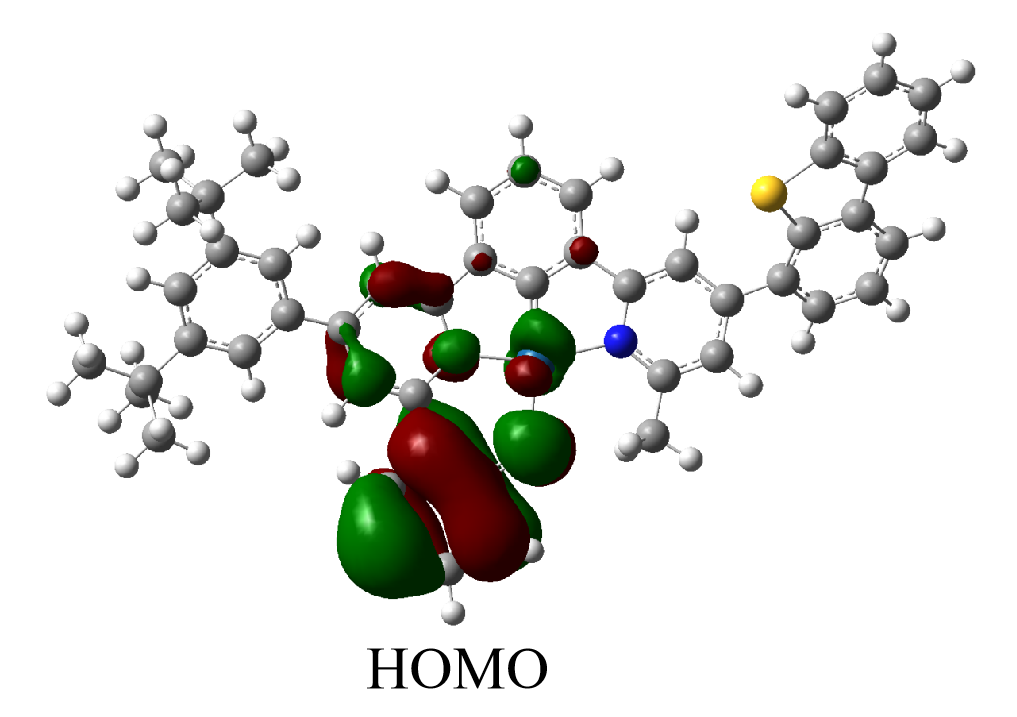


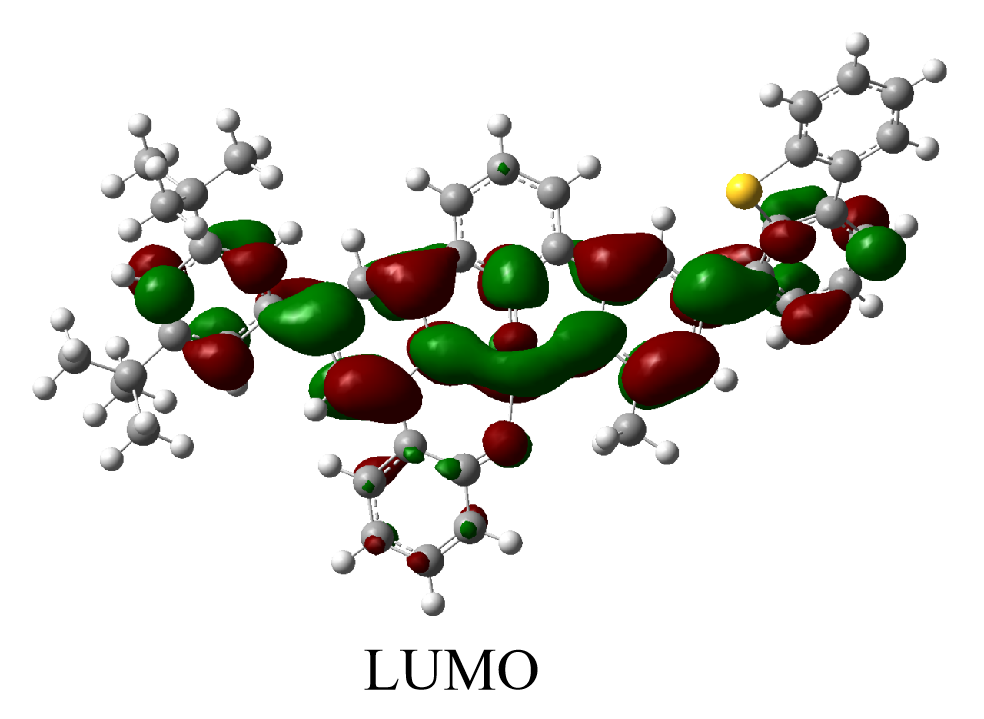


**Figure S2**. HOMO and LUMO at T_1_ structure for monomer of **tetra-Pt-dbt**.


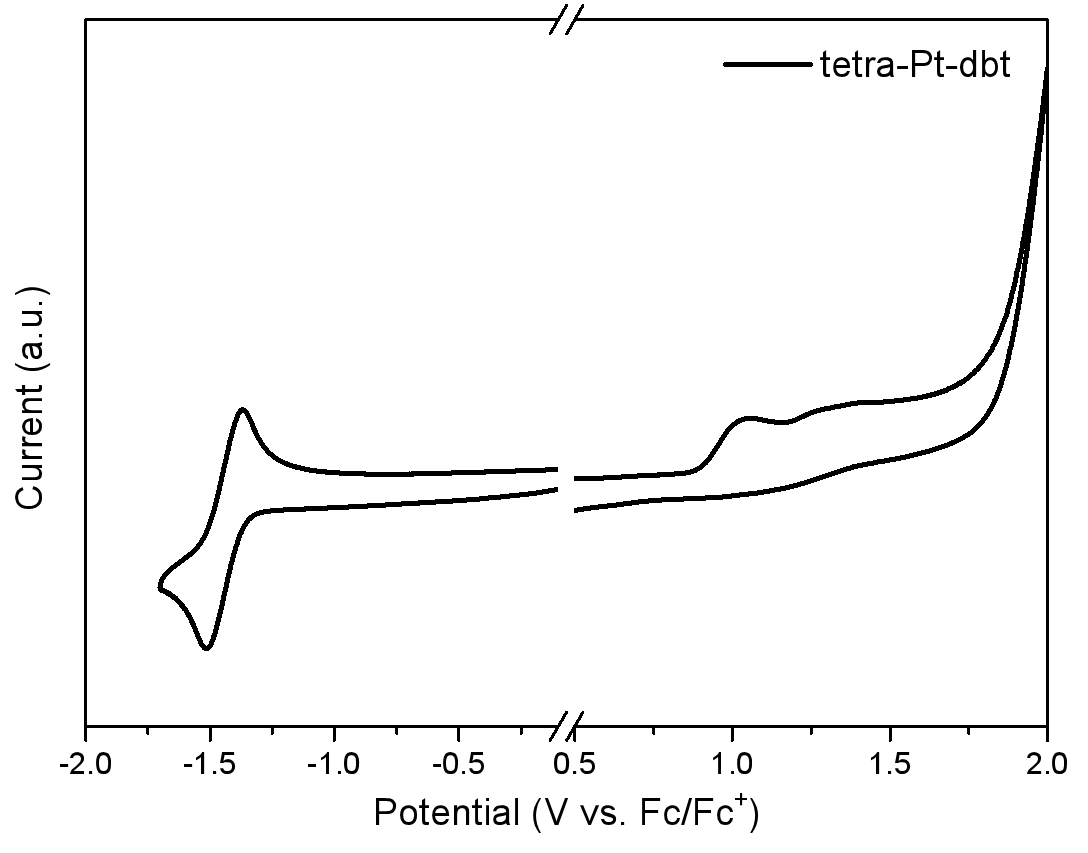


**Figure S3**. Cyclic voltammogram of **tetra-Pt-dbt** in DMF.


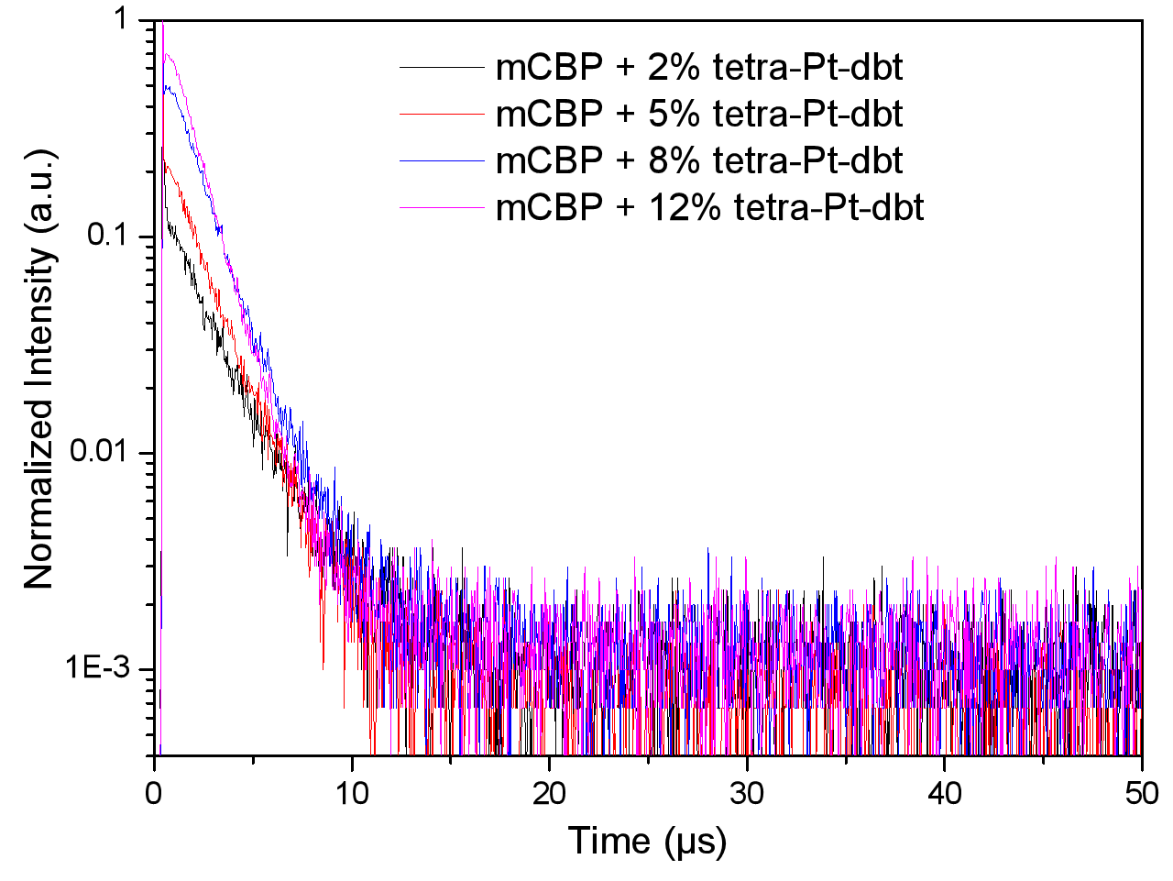


**Figure S4**. Transient PL decay spectra of **tetra-Pt-dbt** in mCBP films with different doping concentrations (2 to 12 wt%) at ~660 nm.

**Table S3.** Selected performances of Device I with different concentrations of **tetra-Pt-dbt** and **tetra-Pt-dbf**.

|  | **Conc. [%]** | Turn-on voltage^a)^  [V] | L ^b)^  [cd m^-2^] | EQE [%] | | CE  [cd A^-1^] | | PE  [lm W^-1^] | | CIE (x,y) | |
| --- | --- | --- | --- | --- | --- | --- | --- | --- | --- | --- | --- |
|  |  |  |  | Max. | at 1000 cd m^-2^ | Max. | at 1000 cd m^-2^ | Max. | at 1000 cd m^-2^ | at 2.6 V | at 7.0 V |
| **tetra-Pt-dbt** | 1 wt% | 2.4 | 106800 | 23.9 | 23.7 | 83.8 | 83.3 | 101.3 | 93.5 | (0.292, 0.636) | (0.283, 0.607) |
|  | 4 wt% | 2.4 | 126400 | 25.5 | 24.9 | 67.4 | 66.9 | 76.0 | 75.1 | (0.365, 0.595) | (0.328, 0.609) |
|  | 6 wt% | 2.4 | 121100 | 23.9 | 23.8 | 51.4 | 51.3 | 55.2 | 53.8 | (0.416, 0.555) | (0.362, 0.587) |
|  | 8 wt% | 2.4 | 106700 | 24.5 | 24.1 | 42.6 | 42.0 | 45.0 | 44.0 | (0.458, 0.519) | (0.391, 0.567) |
| **tetra-Pt-dbf** | 1 wt% | 2.4 | 107600 | 20.9 | 20.4 | 71.9 | 70.6 | 83.9 | 73.4 | (0.306, 0.631) | (0.294, 0.608) |
|  | 4 wt% | 2.4 | 114200 | 23.6 | 22.9 | 56.7 | 55.8 | 62.6 | 59.4 | (0.387, 0.578) | (0.343, 0.599) |
|  | 6 wt% | 2.4 | 90830 | 23.3 | 22.8 | 45.6 | 45.1 | 48.6 | 47.2 | (0.431, 0.543) | (0.373, 0.583) |
|  | 8 wt% | 2.4 | 109000 | 23.1 | 22.7 | 40.2 | 39.0 | 41.6 | 40.9 | (0.457, 0.520) | (0.387, 0.569) |

^a)^ Voltage at the luminance of 1.0 cd m^-2^. ^b)^ Luminance at 7.0 V.


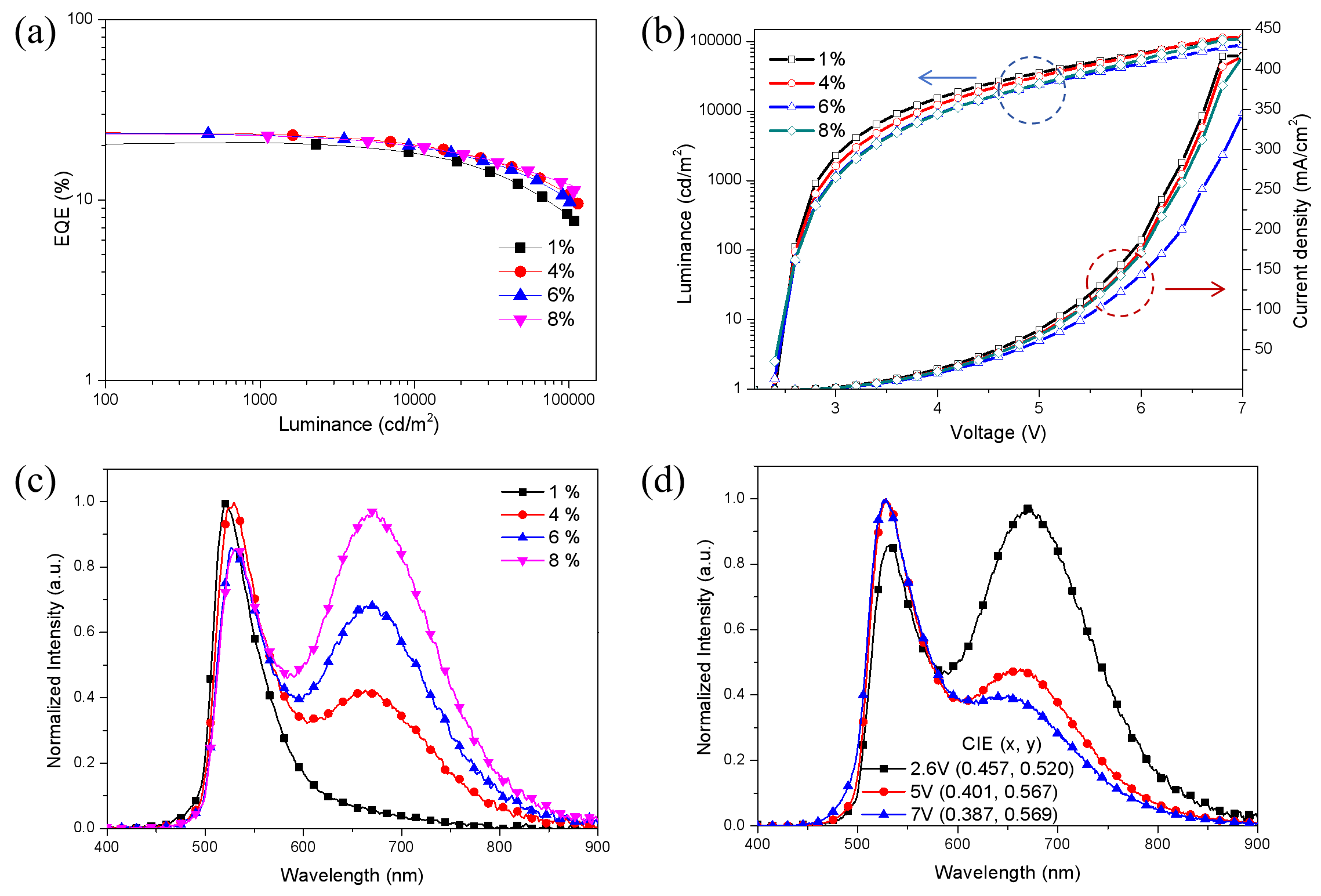


**Figure S5**. (a) External quantum efficiency (EQE) vs. luminance characteristic of the device I with different doping concentrations of **tetra-Pt-dbf**, (b) The luminance-voltage-current density curves of **tetra-Pt-dbf** in Device I, (c) Normalized EL spectra of Device I with different concentrations of **tetra-Pt-dbf** at 2.6V, (d) Normalized EL spectra of Device I with 8 wt% **tetra-Pt-dbf** at 2.6V, 5.0V and 7.0V.


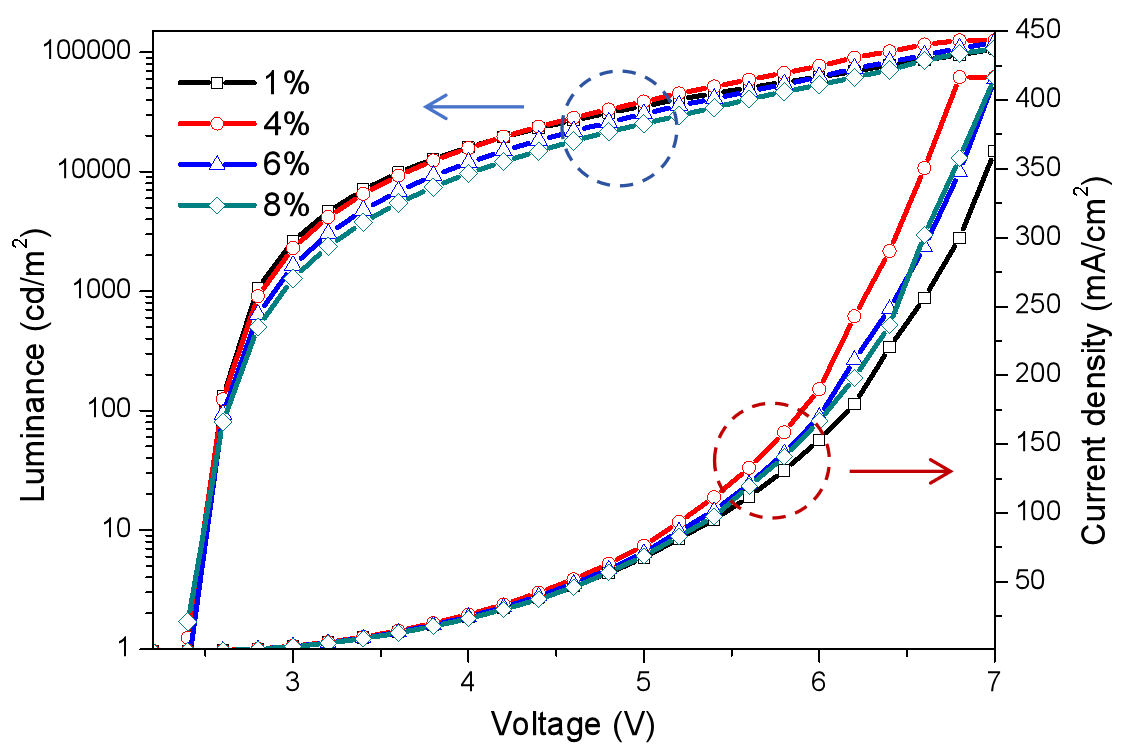


**Figure S6.** The luminance-voltage-current density curves of **tetra-Pt-dbt** in Device I.


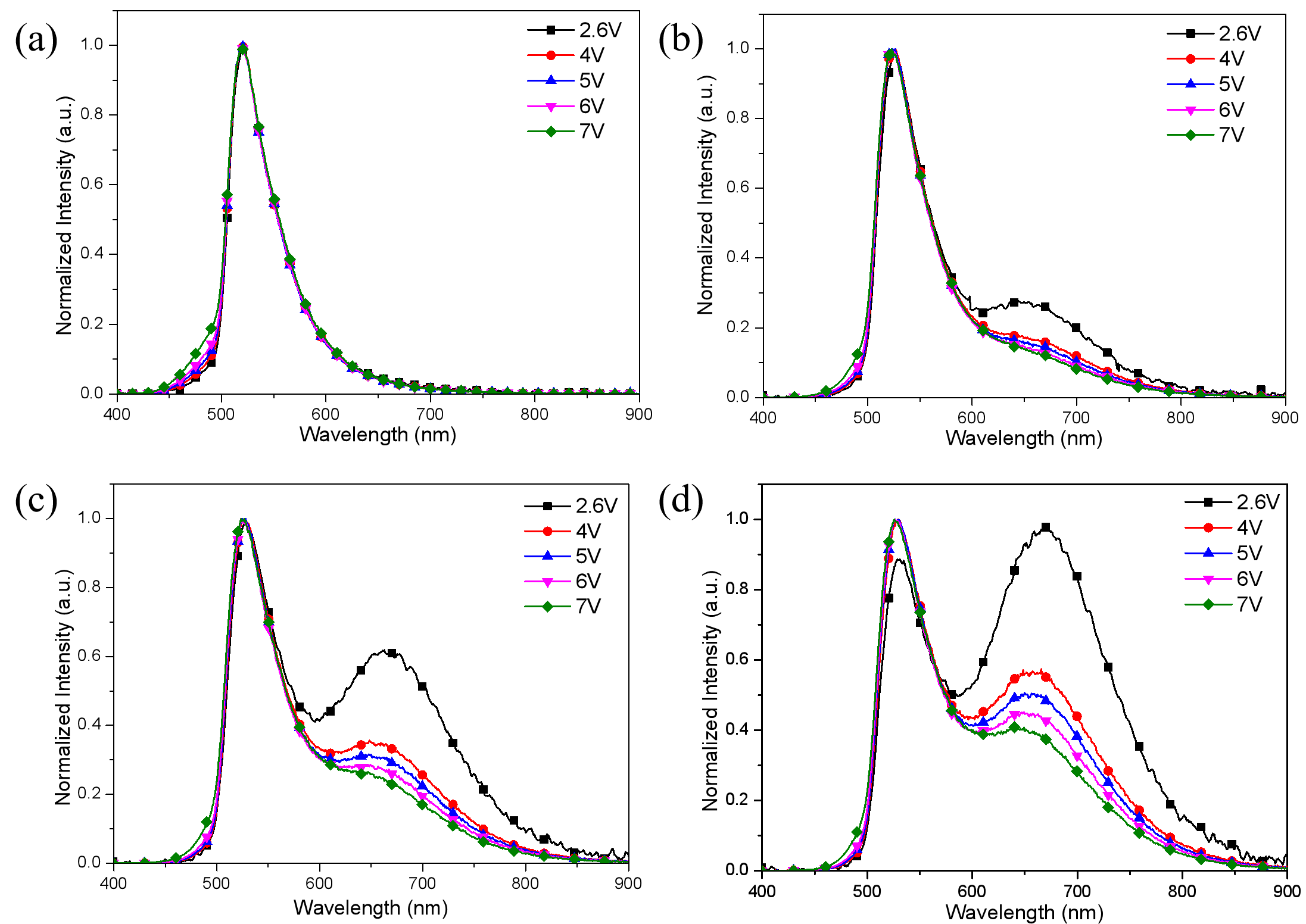


**Figure S7**. Normalized EL spectra of Device I with a) 1 wt%, b) 4 wt%, d) 6 wt%, e) 8 wt% dopant concentration of **tetra-Pt-dbt** at the different voltage from 2.6V to 7.0V.


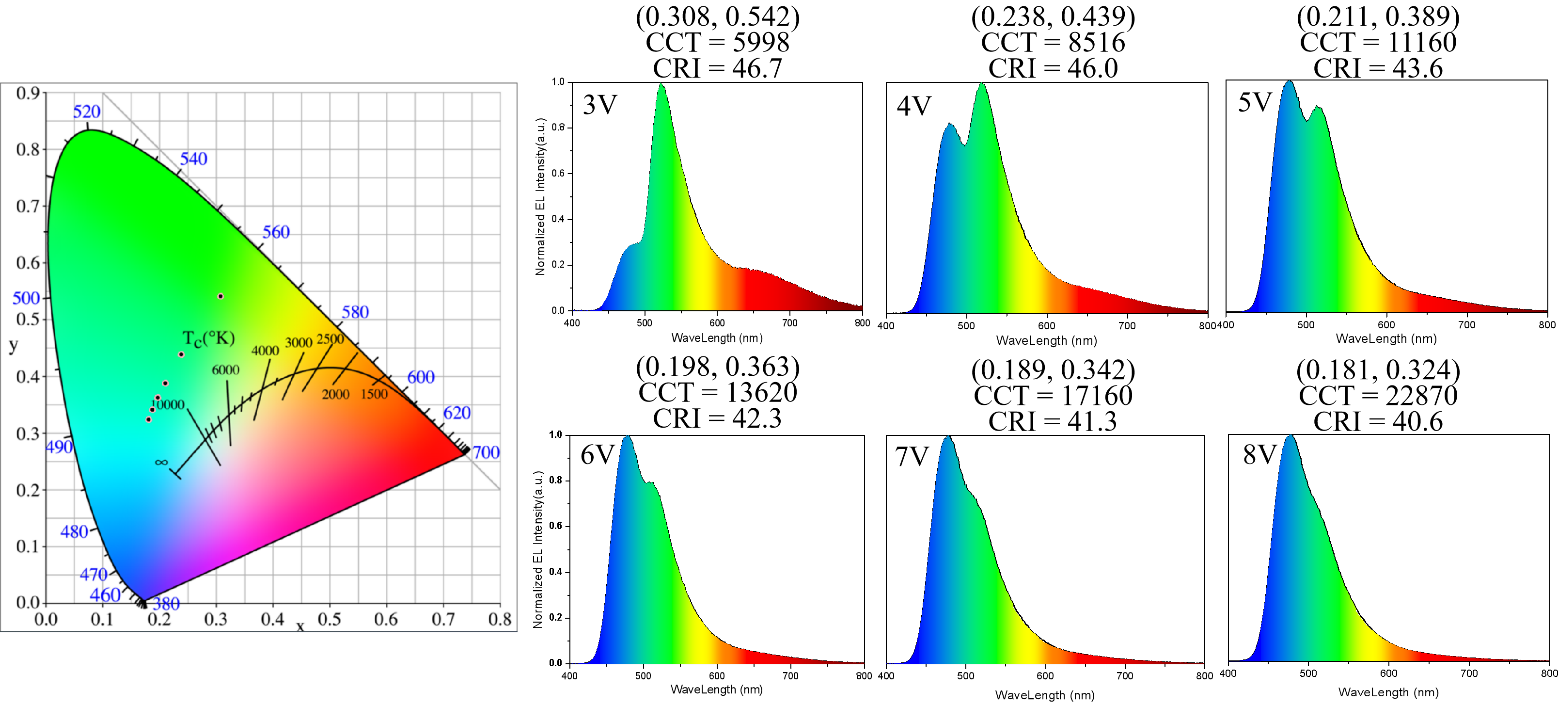


**Figure S8.** CIE coordinates and emission spectra of W1 recorded at 3V, 4V, 5V, 6V, 7V and 8V.


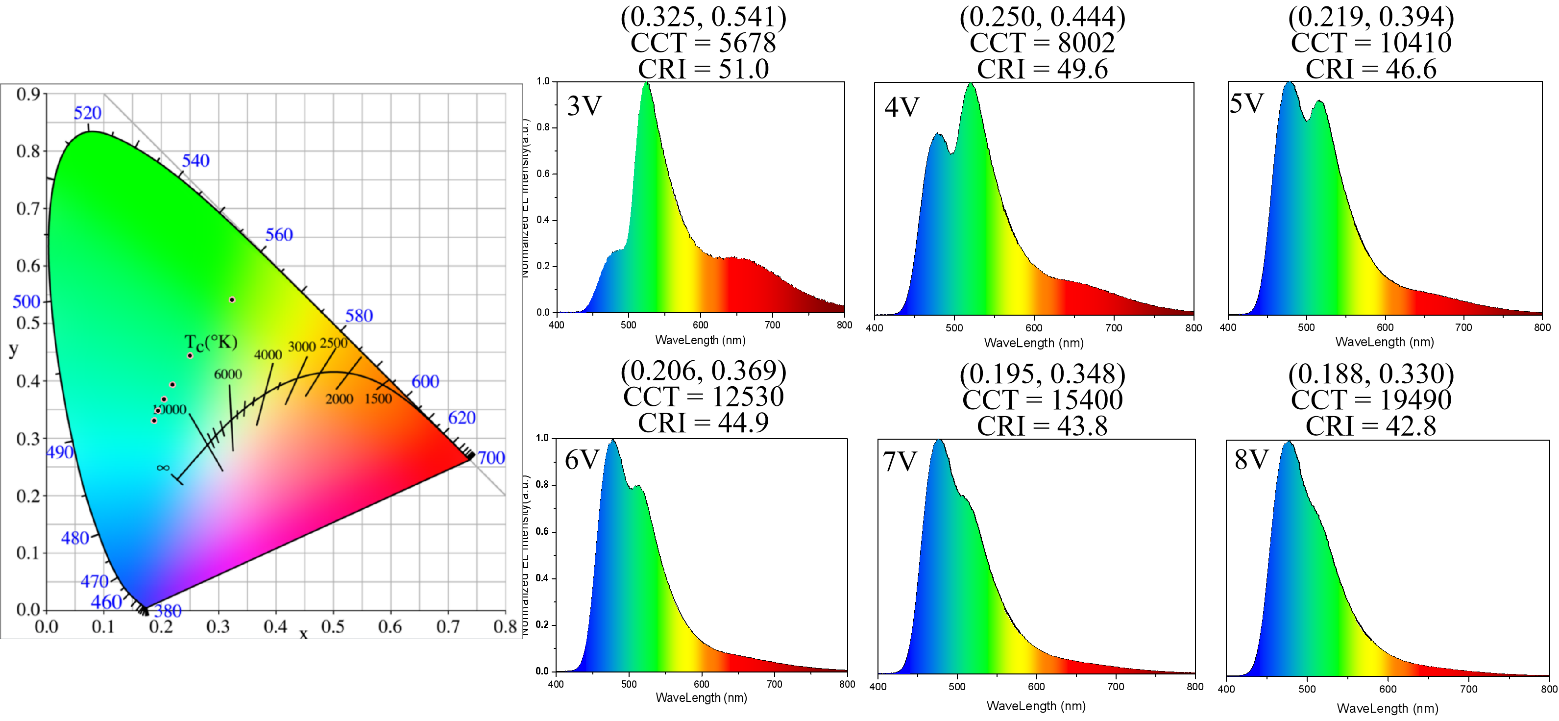


**Figure S9.** CIE coordinates and emission spectra of W2 recorded at 3V, 4V, 5V, 6V, 7V and 8V.


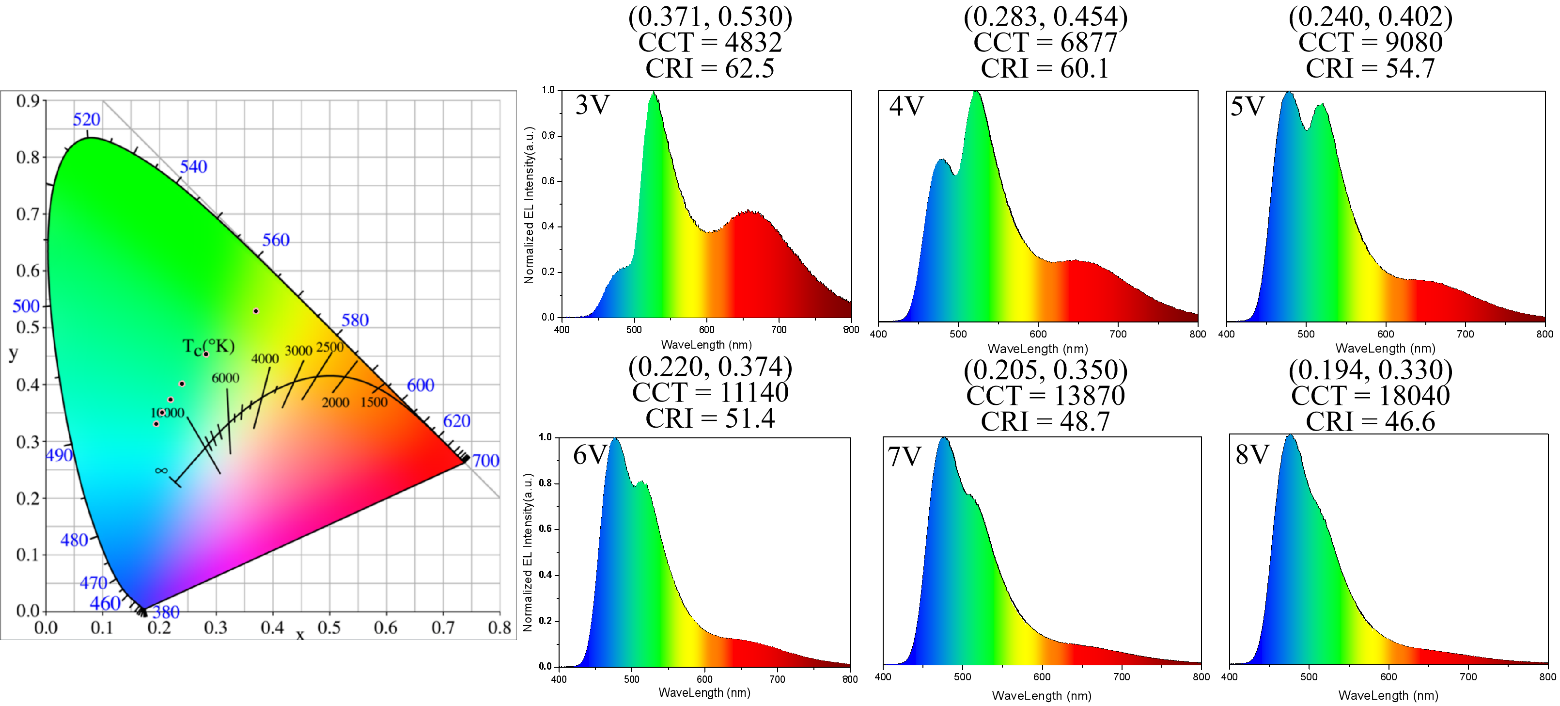


**Figure S10.** CIE coordinates and emission spectra of W3 recorded at 3V, 4V, 5V, 6V, 7V and 8V.


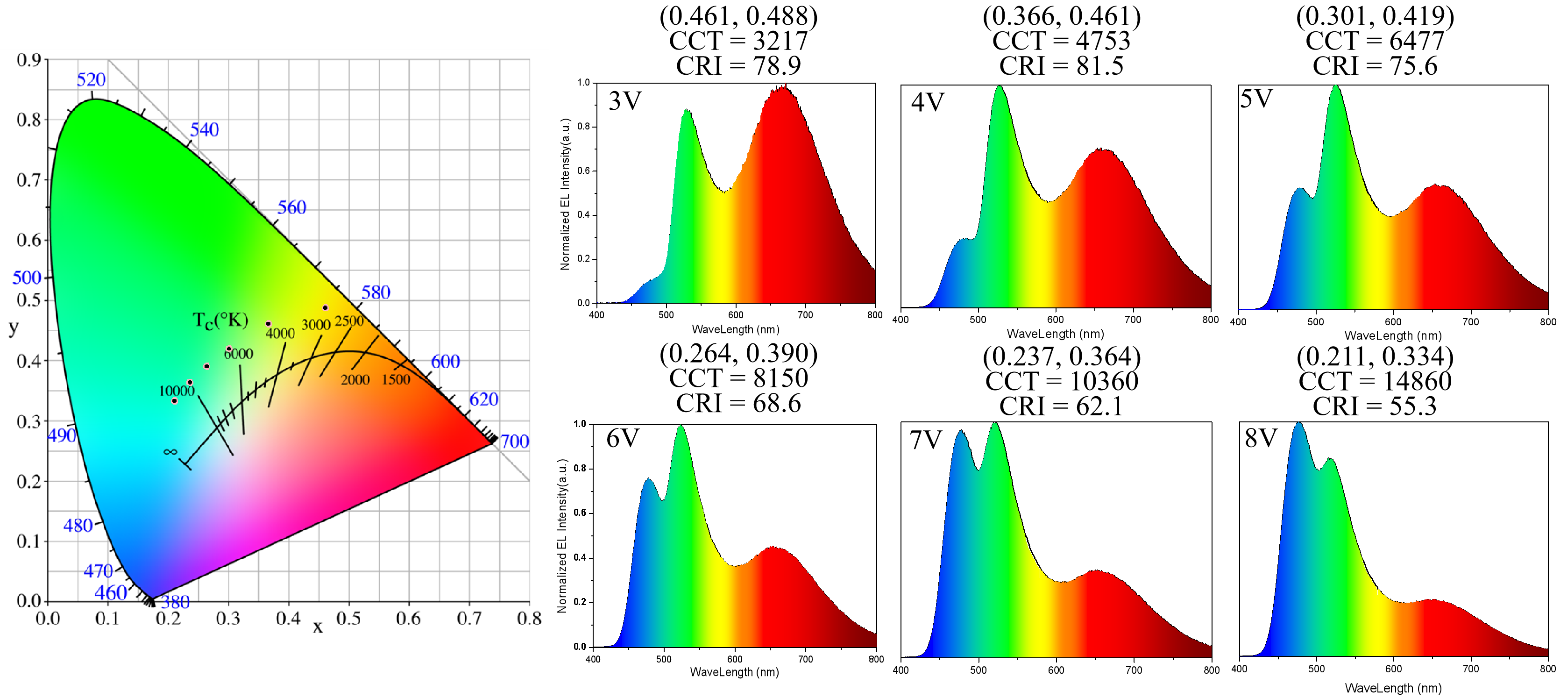


**Figure S11.** CIE coordinates and emission spectra of W4 recorded at 3V, 4V, 5V, 6V, 7V and 8V.


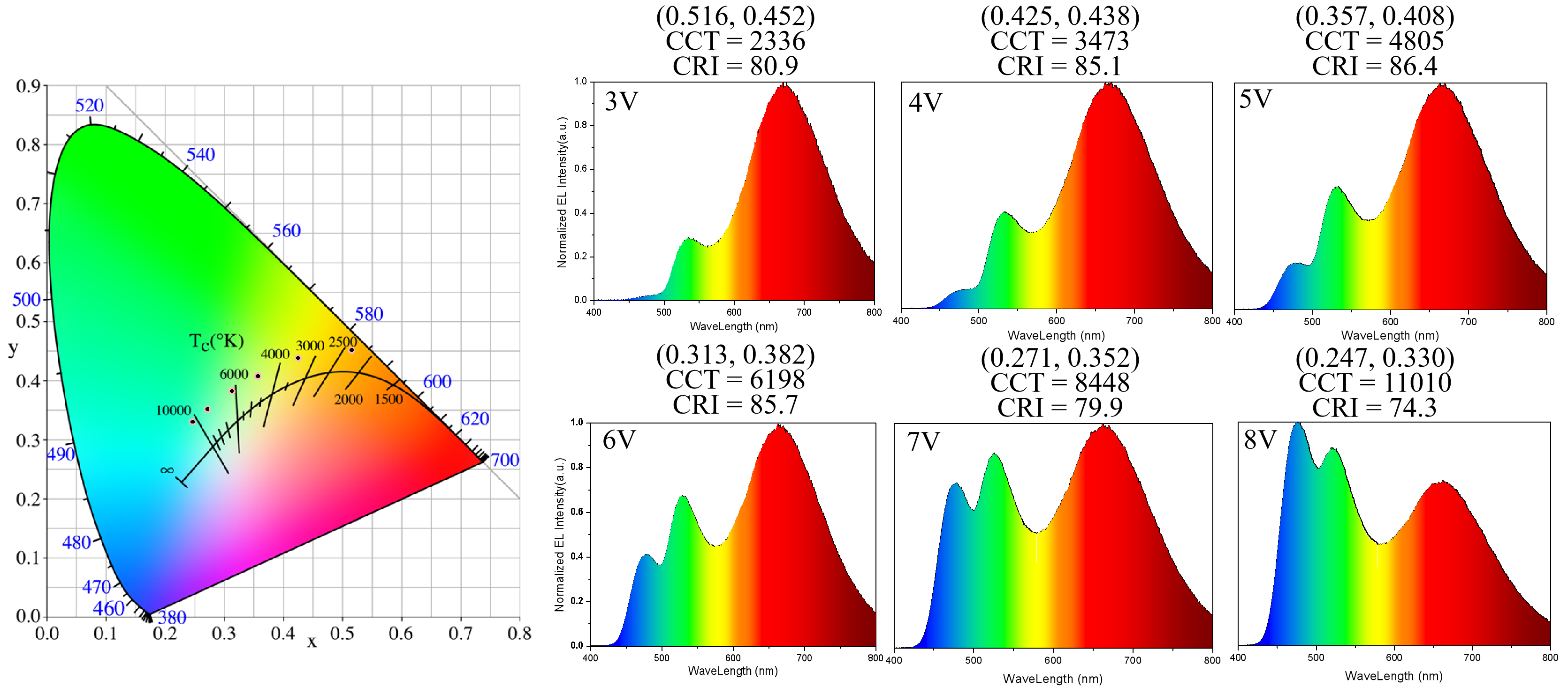


**Figure S12.** CIE coordinates and emission spectra of W5 recorded at 3V, 4V, 5V, 6V, 7V and 8V.

**Table S4**. Representative examples of reported CT-WOLEDs.

| CT-WOLED No. | Emitter(s) | EQE_max_  [%] | CRI | Device lifetime@  luminance (cd m^-2^)  (hrs) | Ref. |
| --- | --- | --- | --- | --- | --- |
| 1 | 4TCzBN-TPh /  **tetra-Pt-dft** | 28.8 | **81.5** | **LT_90_ (100 cd m^-2^) = 21 144 h** | This Work |
| 2 | TDBA-DI/  DACz-AZTRZ | 30.3 | 76.4 | LT_80_ (100 cd m^-2^) = 20 147 | Adv. Mater. 2022, 34, 2103102 |
| 3 | Pt-X-2 | 20.75 | 82.0 |  | Adv. Mater. 2021, 33, 2004873 |
| 4 | PCzT5-Q0.1 | 13.0 | 70.0 | - | Adv. Optical Mater. 2024, 12, 2303067 |
| 5 | PICZ2F/  4CzTPNBu | 23.7 | 73.3 | - | Adv. Optical Mater. 2024, 2400503 |
| 6 | Im2PhPtCl | 24.4 | 83.0 | - | *ACS Materials Lett.* 2023, 5, 920−927 |
| 7 | Ir(mphq)2(acac)/  Ir(ppy)2acac/  Tb-AND+DSB | 18.8 | 70.3 | LT_50_ (1000 cd m^-2^) = 11 204 h | J. Mater. Chem. C, 2019, 7, 15322--15334 |
| 8 | PTZ-DBTO2/DBT | 10.4 | 77.0 | - | Adv. Optical Mater. 2017, 5, 1700435 |
| 9 | Ir(dhfpy)2 (acac)/  TBPe | 5.5 | 68.0 | - | Light: Science & Applications (2016) 5, e16121 |
| 10 | Ir(dhfpy)2 (acac)/  TBPe | 16.0 | 38.0 | - | Light: Science & Applications (2015) 4, e247 |
| 11 | (fbi)2Ir(acac)/FIrpic | 19.3 | - | - | Adv. Funct. Mater. 2009, 19, 84–95 |

**Reference.**

1. F. Wang, T. Z., *J. Chem. Phys.* **2005**, 123.

2. G. te Velde, F. M. B., E. J. Baerends, C. Fonseca Guerra, S. J. A. van Gisbergen, J. G. Snijders, T. Ziegler, *J. Comput. Chem.* **2001**, 22, 931.

3. E.J. Baerends, A. J. A. T. Z., J. Autschbach, O. Baseggio, D. Bashford, A. Bérces, F.M. Bickelhaupt, C. Bo, P.M. Boerrigter, L. Cavallo, C. Daul, D.P. Chong, D.V. Chulhai, L. Deng, R.M. Dickson, J.M. Dieterich, D.E. Ellis, M. van Faassen, L. Fan, T.H. Fischer, C. Fonseca Guerra, M. Franchini, A. Ghysels, A. Giammona, S.J.A. van Gisbergen, A. Goez, A.W. Götz, J.A. Groeneveld, O.V. Gritsenko, M. Grüning, S. Gusarov, F.E. Harris, P. van den Hoek, Z. Hu, C.R. Jacob, H. Jacobsen, L. Jensen, L. Joubert, J.W. Kaminski, G. van Kessel, C. König, F. Kootstra, A. Kovalenko, M.V. Krykunov, E. van Lenthe, D.A. McCormack, A. Michalak, M. Mitoraj, S.M. Morton, J. Neugebauer, V.P. Nicu, L. Noodleman, V.P. Osinga, S. Patchkovskii, M. Pavanello, C.A. Peeples, P.H.T. Philipsen, D. Post, C.C. Pye, H. Ramanantoanina, P. Ramos, W. Ravenek, J.I. Rodríguez, P. Ros, R. Rüger, P.R.T. Schipper, D. Schlüns, H. van Schoot, G. Schreckenbach, J.S. Seldenthuis, M. Seth, J.G. Snijders, M. Solà, M. Stener, M. Swart, D. Swerhone, V. Tognetti, G. te Velde, P. Vernooijs, L. Versluis, L. Visscher, O. Visser, F. Wang, T.A. Wesolowski, E.M. van Wezenbeek, G. Wiesenekker, S.K. Wolff, T.K. Woo, A. L. Yakovlev, ADF2019, SCM, Theoretical Chemistry, Vrije Universiteit, Amsterdam, The Netherlands **2019**.

4. E. van Lenthe, E. J. B., J. G. Snijders, *J. Chem. Phys.* **1993**, 99, 4597.

5. E. van Lenthe, E. J. B., J. G. Snijders, *J. Chem. Phys.* **1994**, 101, 9783.

6. E. van Lenthe, A. E., E.-J. Baerends, *J. Chem. Phys*. **1999**, 110, 8943.

7. Zhao, Y., Truhlar, D. G., *Theor. Chem. Acc.* **2008**, 120 (1), 215-241.

8. Barbieri, P. L., Fantin, P. A., Jorge, F. E., *Mol. Phys*. **2006**, 104 (18), 2945-2954.

9. Machado, S. F., Camiletti, G. G., Neto, A. C., Jorge, F. E., Jorge, R. S., *Mol. Phys*. **2009**, 107 (16), 1713-1727.

10. Klamt, A., *J. Phys. Chem.* **1995**, 99 (7), 2224-2235.

11. A. Klamt, G. S., J. Chem. Soc., *Perkin Trans.* 2 **1993**, 799.

12. Grimme, S., Antony, J., Ehrlich, S., Krieg, H., *J. Chem. Phys.* **2010**, 132 (15), 154104.

13. Frisch, M. J., Pople, J. A., Binkley, J. S., *J. Chem. Phys.* **1984**, 80 (7), 3265-3269.

14. Radom, L., Hariharan, P. C., Pople, J. A. *J. Am. Chem. Soc.‌‌* **1973**, 95 (20), 6531-6544.

15. Hay, P. J., Wadt, W. R., *J. Chem. Phys.* **1985**, 82 (1), 299-310.

16. Wadt, W. R., Hay, P. J., *J. Chem. Phys.* **1985**, 82 (1), 284-298.

17. Cossi M., calmani, G. Rega, N., Barone, V., *J. Chem. Phys.* **2002**, 117 (1), 43-54.

18. te Velde, G., Bickelhaupt, F. M., Baerends, E. J., Fonseca Guerra, C., van Gisbergen, S. J. A., Snijders, J. G., Ziegler, T., *J. Comput. Chem.* **2001**, 22 (9), 931-967.

19. Lenthe, E. v., Baerends, E. J., Snijders, J. G., *J. Chem. Phys****.* 1993**, 99 (6), 4597-4610.

20. van Lenthe, E., Baerends, E. J., Snijders, J. G., *J. Chem. Phys.* **1994**, 101 (11), 9783-9792.

21. van Lenthe, E., Ehlers, A., Baerends, E.-J., *J. Chem. Phys.***1999**, 110 (18), 8943-8953.
